# Supplementary material for: D-O-A based organic phosphors for both aggregation-induced electrophosphorescence and host-free sensitization
Source: Nat Commun. 2023 Mar 25;14:1678. doi: 10.1038/s41467-023-37414-y (PMC10039947; doi:10.1038/s41467-023-37414-y)
Supplement: Supplementary file 1 — Supplementary Information [file 41467_2023_37414_MOESM1_ESM.pdf]

SUPPLEMENTARY INFORMATION

D-O-A based organic phosphors for both aggregation-induced electrophosphorescence and host-free sensitization

Supplementary methods

Synthesis: All reagents were purchased from commercial channels, and used directly unless specifically mentioned. *N*,*N*-dimethylformamide (DMF), toluene and *N*-methyl pyrrolidone (NMP) were purified according to the standard procedures. 2-(4-fluorophenyl)-4,6-diphenyl-1,3,5-triazine was purchased from Shanghai Haoyuan Chem express Co. Ltd. with a purity of 98%.

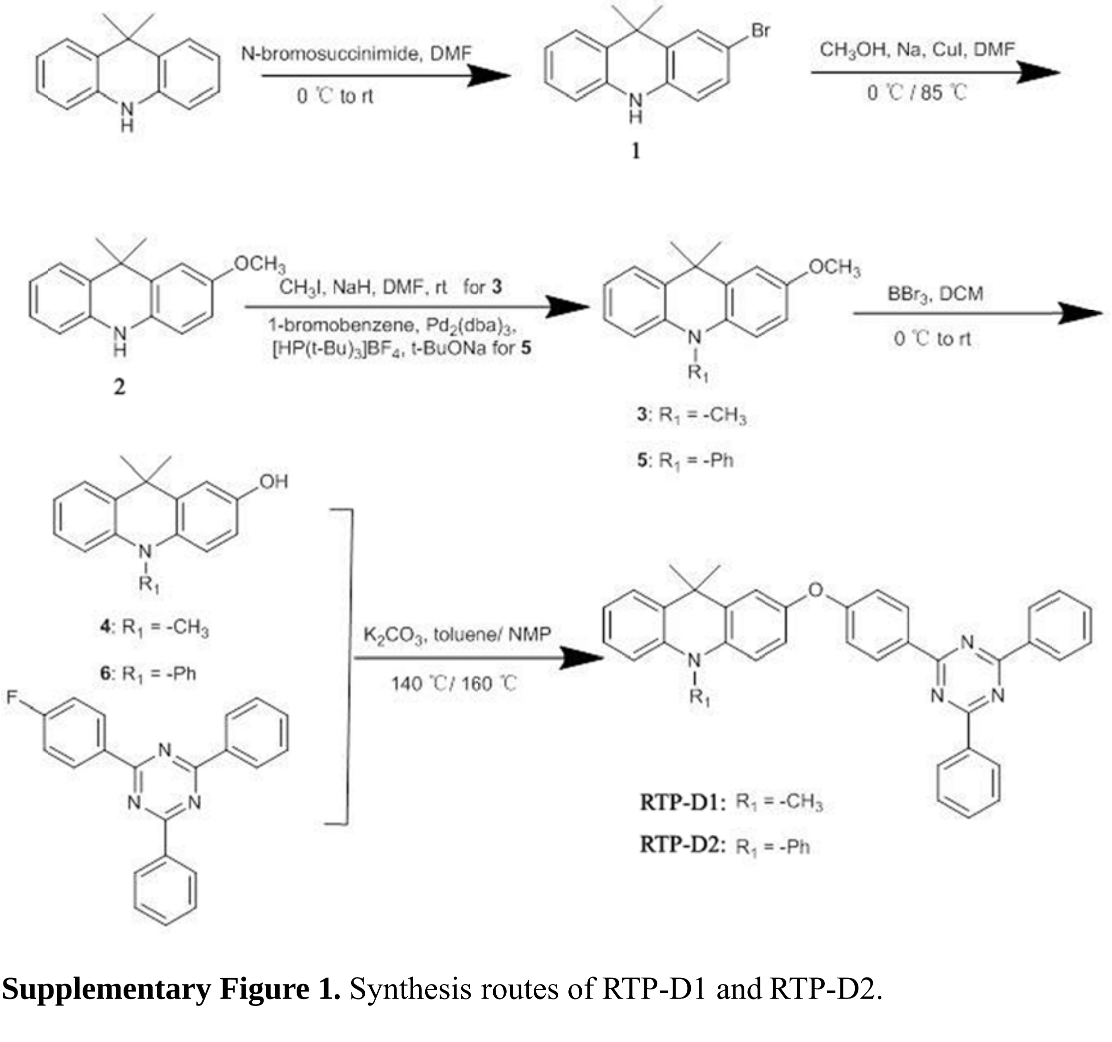

Supplementary Figure 1. Synthesis routes of RTP-D1 and RTP-D2.

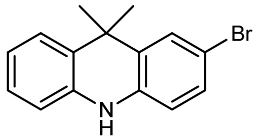

2-Bromo-9,10-dihydro-9,9-dimethylacridine (**1**): N-bromosuccinimide (18.60 g, 105.12 mmol) was slowly added into a solution of 9,9-dimethyl-9,10-dihydroacridine (20.90 g, 99.94 mmol) in 120 mL dry DMF at 0 ^{o}C. Then the system was stirred for 2 h at room temperature. After addition of 300 mL water, the mixture was extracted with dichloromethane, and washed with aqueous brine. The separated organic layer was dried over sodium sulfate, filtered and purified by column chromatography on silica gel using petroleum ether/dichloromethane (v/v 5:1) as the eluent to give a white solid **1** (19.00 g, 93%). ^{1}H NMR (400 MHz, $CDCl_3$, δ ppm): 7.47 (d, *J* = 2.1 Hz, 1H), 7.40 (dd, *J* = 7.8, 0.9 Hz, 1H), 7.20 (dd, *J* = 8.4, 2.1 Hz, 1H), 7.13 (td, *J* = 7.8, 1.3 Hz, 1H),, 6.95 (t, *J* = 7.4 Hz, 1H), 6.70 (d, *J* = 7.8 Hz, 1H), 6.58 (d, *J* = 8.4 Hz, 1H), 6.16 (brs, 1H), 1.57 (s, 6H). ^{13}C NMR (100 MHz, $CDCl_3$): 138.0, 137.6, 137.1, 131.3, 130.7, 129.7, 129.5, 128.5, 127.0, 125.5, 121.1, 115.0, 113.6, 36.4, 30.5. HR-ESI-MS (*m/z*): $[M+H]^+$ calcd. for C15H15BrN, 288.0382; Found, 288.0385.

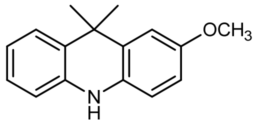

9,10-Dihydro-2-methoxy-9,9-dimethylacridine (**2**): Sodium lumps (7.97 g, 346.81 mmol) were cut up and slowly added in a 250 mL round-bottom flask containing 100 mL dry methanol under argon atmosphere. After dissolved completely, the mixture was transferred into a flame-dried two-necked round-bottom flask with a solution of

2-bromo-9,10-dihydro-9,9-dimethylacridine (**1**) (9.01 g, 31.39 mmol) and CuI (17.80 g, 93.46 mmol) in 150 mL dry DMF. The reaction mixture was heated at 85 °C for 10 h. When cooled to room temperature, the system was filtered, poured into water and extracted by dichloromethane. After removing the solvent, the crude produce was purified by silica gel column chromatography to give a white crystal **2** (3.71 g, 56%). ^{1}H NMR (400 MHz, DMSO-d_6, δ ppm): 8.62 (s, 1H), 7.30 (d, *J* = 7.5 Hz, 1H), 7.01 (t, *J* = 8.0 Hz, 1H), 6.91 (d, *J* = 2.4 Hz, 1H), 6.70 - 6.77 (m, 4H), 3.70 (s, 3H), 1.47 (s, 6H). ^{13}C NMR (100 MHz, DMSO-d_6): 153.3, 139.4, 132.9, 129.3, 127.2, 126.6, 125.5, 119.1, 114.0, 113.2, 112.3, 111.3, 55.4, 36.0, 30.8. HR-ESI-MS (*m/z*): $[M+H]^+$ calcd. for C16H18NO, 240.1383; Found, 240.1383.

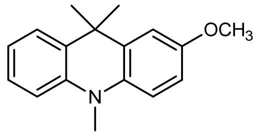

9,10-Dihydro-2-methoxy-9,9-dimethyl-10-methylacridine (**3**): A mixture of 9,10-dihydro-2-methoxy-9,9-dimethylacridine (**2**) (2.21 g, 9.24 mmol) and sodium hydride (0.35 g, 14.72 mmol) and dry THF (60 mL) was stirred at room temperature for 1h. Then iodomethane (1.57 g, 11.04 mmol) was injected into the mixture, and reacted for 6 h. The mixture was slowly poured into 150 mL water, and extracted three times with dichloromethane. And a colorless transparent oily liquid **3** (2.28 g, 98%) was obtained after removing the solvent. ^{1}H NMR (400 MHz, $CDCl_3$, δ ppm): 7.31 (dd, *J* = 7.7, 1.6 Hz, 1H), 7.12-7.16 (m, 1H), 6.93 (d, *J* = 2.8 Hz, 1H), 6.88 (td, *J* = 7.5, 1.0 Hz, 1H), 6.82 (d, *J* = 8.1 Hz, 1H), 6.77 (d, *J* = 8.7 Hz, 1H), 6.69 (dd, *J* = 8.7,

2.8 Hz, 1H) 3.72 (s, 3H), 3.32 (s, 3H), 1.44 (s, 6H). ^{1}H NMR (100 MHz, $CDCl_3$): 154.2, 136.6, 134.3, 132.0, 126.6, 123.6, 120.1, 112.4, 111.7, 110.8, 110.5, 55.7, 36.7, 33.3, 27.0. HR-ESI-MS (*m/z*): $[M+H]^+$ calcd. for C17H20NO, 254.1539; Found, 254.1537.

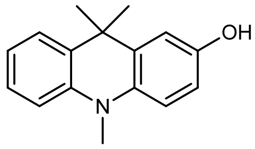

9,10-Dihydro-9,9-dimethyl-10-methyl-2-acridinol (**4**): Boron tribromide (0.67 mL, 18.04 mmol) was injected into a 100 mL two-necked round-bottom flask containing a solution of 9,10-Dihydro-2-methoxy-9,9-dimethyl-10-methylacridine (**3**) (2.28 g, 9.02 mmol) in dry dichloromethane (45 mL) at 0 °C. After stirred at room temperature for 4 h, the mixture was slowly poured into 100 mL water and extracted three times with dichloromethane. After removing the solvent, the crude produce was purified by silica gel column chromatography using petroleum ether/dichloromethane/acetone (v/v/v 100/50/1) as the eluent to afford a white powder **4** (1.92 g, 89%). ^{1}H NMR (400 MHz, DMSO-d_6, δ ppm): 8.84 (s, 1H), 7.35 (d, J = 7.7 Hz, 1H), 7.17 (t, J = 7.7 Hz, 1H), 6.88 - 6.94 (m, 2H), 6.81 - 6.84 (m, 2H), 6.63 (dd, J = 8.7, 2.7 Hz, 1H), 3.32 (s, 3H), 1.41 (s, 6H). ^{13}C NMR (100 MHz, DMSO-d_6): 152.2, 142.9, 135.2, 133.8, 131.9, 127.0, 123.9, 120.2, 113.4, 113.2, 112.2, 111.2, 36.6, 33.6, 27.4. HR-ESI-MS (*m/z*): $[M+H]^+$ calcd. for C18H16NO, 240.1383; Found, 240.1382.

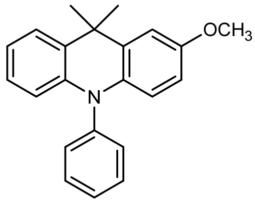

9,10-Dihydro-2-methoxy-9,9-dimethyl-10-phenylacridine (**5**): Under argon atmosphere, 1-bromobenzene (0.69 mL, 6.56 mmol) was poured into a 100 mL two-necked round-bottom flask containing tris (dibenzylideneacetone) dipalladium (0.20 g, 0.22 mmol), tri-tert-butylphosphine tetrafluoroborate (0.32 g, 1.10 mmol), sodium tert-butoxide (1.31 g, 13.67 mmol), 9,10-dihydro-2-methoxy-9,9-dimethylacridine (**2**) (1.31 g, 5.47 mmol) and toluene (60 mL). The mixture was stirred and reflux at 115 °C for 10 h. When cooled to room temperature, 180 mL water was added, and the mixture was extracted with dichloromethane for three times. After removing the solvent, the crude produce was purified by silica gel column chromatography using petroleum ether/dichloromethane (v/v 10:1) as the eluent to give a white powder **5** (1.60 g, 96%). ^{1}H NMR (400 MHz, $CDCl_3$, δ ppm): 7.61-7.64 (m, 2H), 7.44-7.44 (m, 2H), 7.34-7.36 (m, 2H), 7.06-7.10 (m, 1H), 6.89-7.00 (m, 2H), 6.55-6.59 (m, 1H), 6.20-6.29 (m, 2H), 3.79 (s, 3H), 1.70 (s, 6H). ^{13}C NMR (100 MHz, $CDCl_3$): 154.0, 141.5, 141.2, 135.4, 131.4, 131.3, 130.8, 129.1, 128.0, 126.3, 125.1, 120.0, 114.5, 113.7, 111.6, 110.9, 55.6, 36.2, 30.8. HR-ESI-MS (*m/z*): $[M+H]^+$ calcd. for C22H22NO, 316.1696; Found, 316.1694.

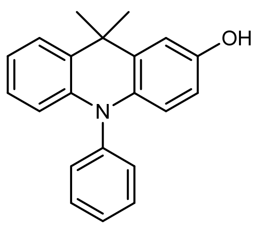

9,10-Dihydro-9,9-dimethyl-10-phenyl-2-acridinol (**6**): According to the same procedure of **4**, the hydroxyl intermediate **6** was synthesized by Boron tribromide (0.43 mL, 11.54 mmol) and 9,10-Dihydro-9,9-dimethyl-10-methyl-2-acridinol (**5**) (1.82 g, 5.77 mmol), and purified by silica gel column chromatography using petroleum ether/ethyl acetate (v/v 30:1) as the eluent to give a white powder (1.62 g, 93%). ^{1}H NMR (400 MHz, DMSO-d_6, δ ppm): 8.89 (s, 1H), 7.67 (t, J = 7.7 Hz, 2H), 7.53 (t, J = 7.4 Hz, 1H), 7.44 (d, J = 7.7 Hz, 1H), 7.33 (d, J = 7.4 Hz, 2H), 6.91-6.95 (m, 2H), 6.84 (td, J = 7.4, 1.0 Hz, 1H), 6.43 (dd, J = 8.8, 2.7 Hz, 1H), 6.12 (d, J = 8.2 Hz, 1H), 5.98 (d, J = 8.8 Hz, 1H), 1.58 (s, 6H). ^{13}C NMR (100 MHz, DMSO-d_6): 152.1, 141.6, 141.4, 133.9, 131.5, 131.3, 129.3, 128.6, 126.8, 125.7, 120.2, 115.0, 113.6, 113.5, 112.3, 36.2, 31.2. HR-ESI-MS (*m/z*): $[M+H]^+$ calcd. for C21H20NO, 302.1539; Found, 547.1537.

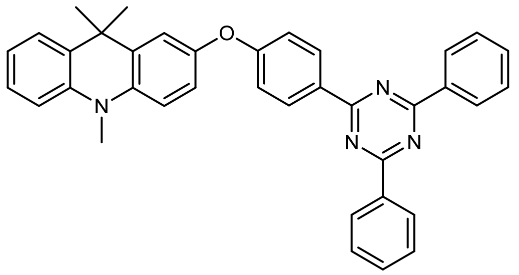

Synthesis of RTP-D1: 9,10-Dihydro-9,9-dimethyl-10-methyl-2-acridinol (**4**: 1.92 g, 8.03 mmol), K_2CO_3 (1.66 g, 12.04 mmol), 15 mL dry NMP and 15 mL dry toluene were added into a 100 mL three-necked round-bottom flask, and refluxed 140 ^{o}C for 2

h under argon atmosphere. When cooled to room temperature, 2-(4-fluorophenyl)-4,6-diphenyl-1,3,5-triazine (2.28 g, 6.98 mmol) dissolved in 15 mL dry NMP was added. The mixture was heated to 160 °C and reacted for another 8 h. After extracted with dichloromethane and removing the solvent, the crude product was purified by silica gel column chromatography using petroleum ether/dichloromethane (v/v 7:1) as the eluent to afford a white powder RTP-D1 (2.36 g, 62%). ^{1}H NMR (500 MHz, $CDCl_3$, δ ppm): 8.75 (m, 6H), 7.59 (m, 6H), 7.43 (dd, *J* = 7.6, 1.5 Hz, 1H), 7.29 (m, 1H), 7.24 (d, *J* = 2.6 Hz, 1H), 7.10 (m, 2H), 6.97-7.05 (m, 4H), 3.50 (s, 3H), 1.56 (s, 6H). ^{13}C NMR (125 MHz, $CDCl_3$, δ ppm): 171.48, 171.13, 162.97, 149.40, 142.25, 139.53, 136.36, 134.71, 132.08, 130.92, 129.95, 128.93, 128.62, 126.81, 123.74, 120.78, 118.43, 116.73, 116.53, 112.99, 112.14, 36.84, 33.58, 27.12. HR-ESI-MS (*m/z*): $[M+H]^+$ calcd. for C37H31N4O, 547.2492; Found, 547.2496. Elemental analysis calcd. (%) for $C_{37}H_{30}N_4O$: C, 81.29; H, 5.53; N, 10.25; Found: C, 81.58; H, 5.23; N, 10.25.

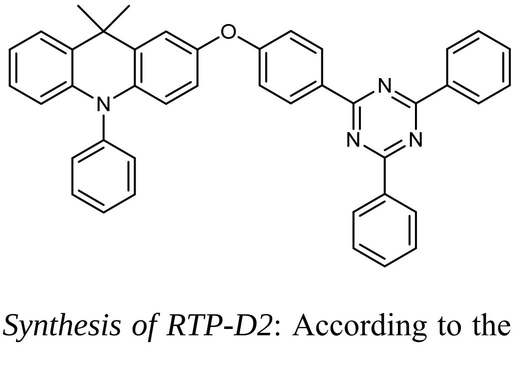

Synthesis of RTP-D2: According to the same procedure of RTP-D1, 15 mL dry NMP and 15 mL dry toluene were added in a 100 mL three-necked round-bottom flask including 9,10-Dihydro-9,9-dimethyl-10-phenyl-2-acridinol (**6**: 1.51 g, 5.02 mmol) and K_2CO_3 (1.04 g, 7.53 mmol) under argon atmosphere, refluxed 140 °C for 2 h.

After cooling to room temperature, 2-(4-fluorophenyl)-4,6-diphenyl-1,3,5-triazine (1.98 g, 4.36 mmol) and 15 mL dry NMP was added. The mixture was heated to 160 ^{o}C overnight. The silica gel column chromatography with petroleum ether/dichloromethane (v/v 7:1) was used to obtain RTP-D2 (white powder, 1.92 g) in a yield of 60%. ^{1}H NMR (500 MHz, DMSO-d_6, δ ppm): 8.71 (d, J = 8.3 Hz, 6H), 7.68 - 7.73 (m, 4H), 7.64 (t, J = 7.8 Hz, 4H), 7.59 (t, J = 7.5 Hz, 1H), 7.49 (dd, J = 7.6, 1.6 Hz, 1H), 7.42 (d, J = 7.4 Hz, 2H), 7.35 (d, J = 2.8 Hz, 1H), 7.13 (d, J = 8.9 Hz, 2H),6.99 (td, J = 7.5, 1.5 Hz, 1H), 6.91 (td, J = 7.5, 1.2 Hz, 1H), 6.86 (dd, J = 8.9, 2.7 Hz, 1H), 6.23 (d, J = 8.9 Hz, 1H), 6.17 (dd, J = 8.3, 1.2 Hz, 1H), 1.64 (s, 6H). ^{13}C NMR (125 MHz, DMSO-d_6, δ ppm): 170.83, 170.44, 162.19, 148.51, 140.58, 140.17, 137.54, 135.39, 132.63, 131.49, 130.92, 130.72, 130.66, 129.19, 128.85, 128.66, 128.42, 128.29, 126.31, 125.09, 120.43, 118.21, 117.27, 116.62, 114.74, 113.45, 35.71, 30.80. HR-ESI-MS (*m/z*): $[M+H]^+$ calcd. for C42H33N4O, 609.2649; Found, 609.2647. Elemental analysis calcd. (%) for $C_{42}H_{32}N_4O$: C, 82.87; H, 5.30; N, 9.20; Found C, 82.58; H, 5.24; N, 8.96.

Supplementary Figures

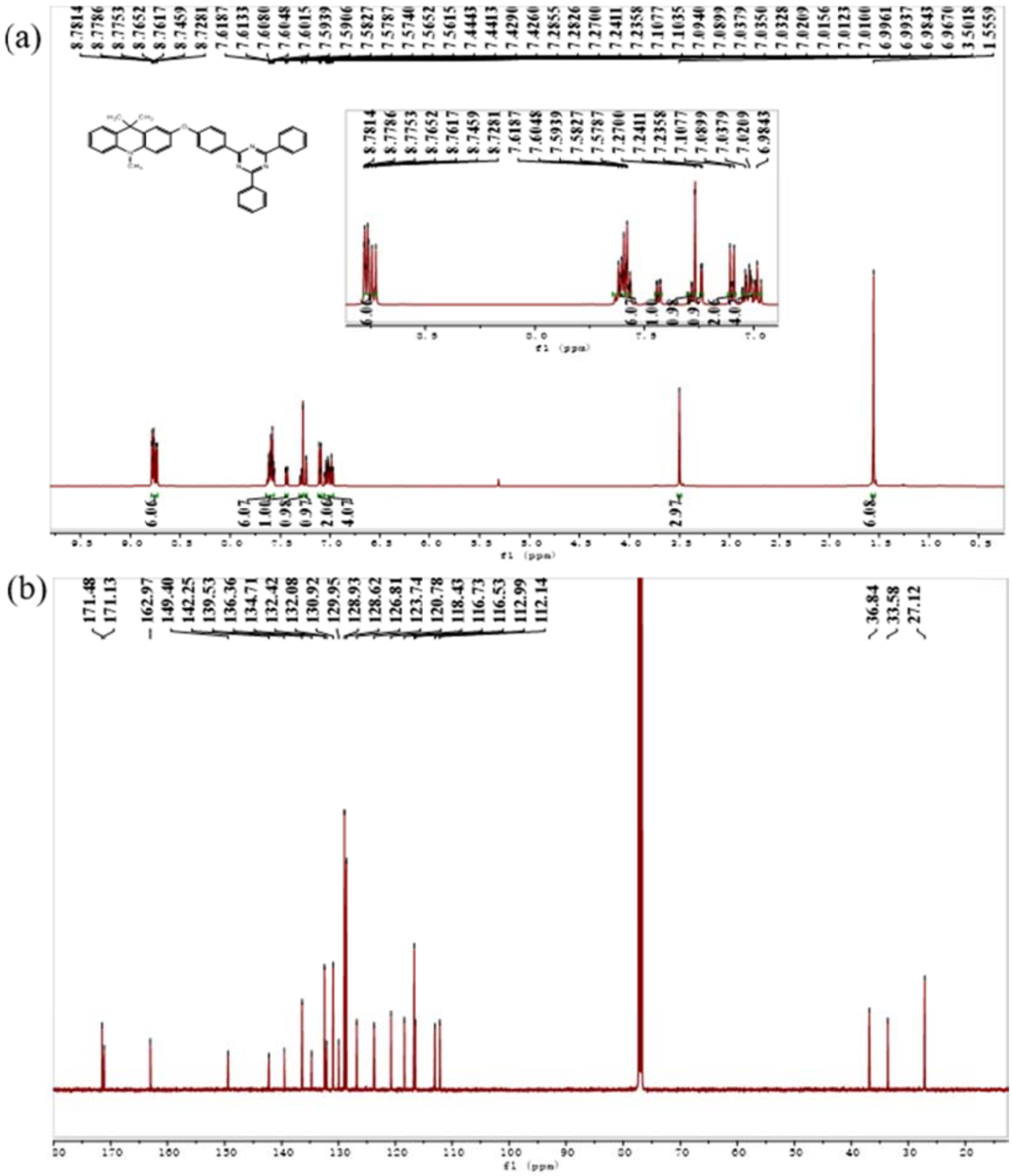

Supplementary Figure 2. ^{1}H (a) and ^{13}C NMR spectra (b) of RTP-D1.

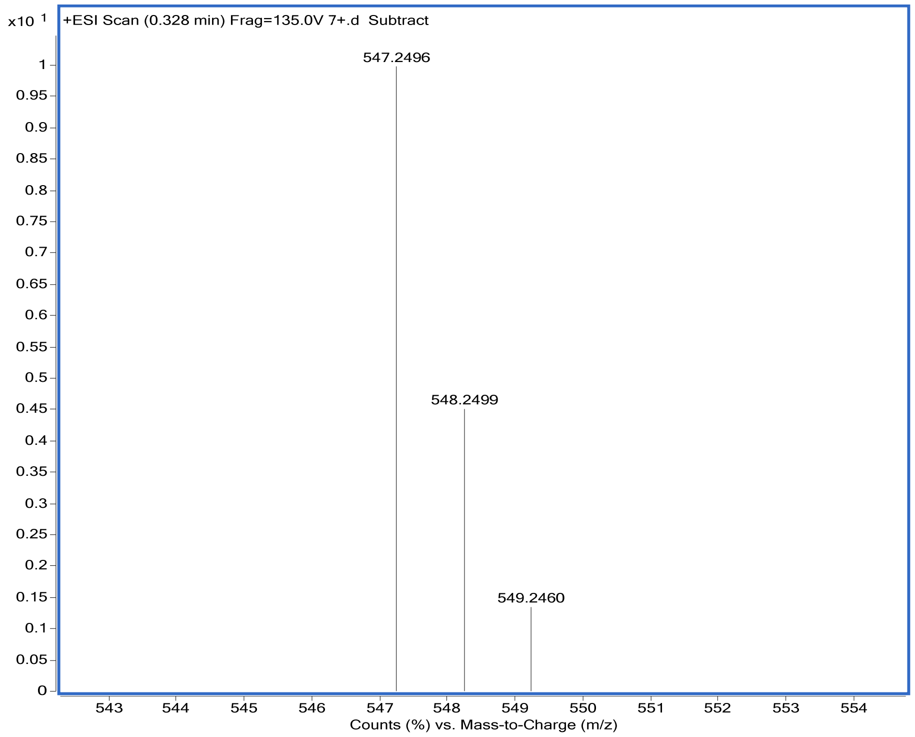

Supplementary Figure 3. HR-ESI-MS spectrum of RTP-D1.

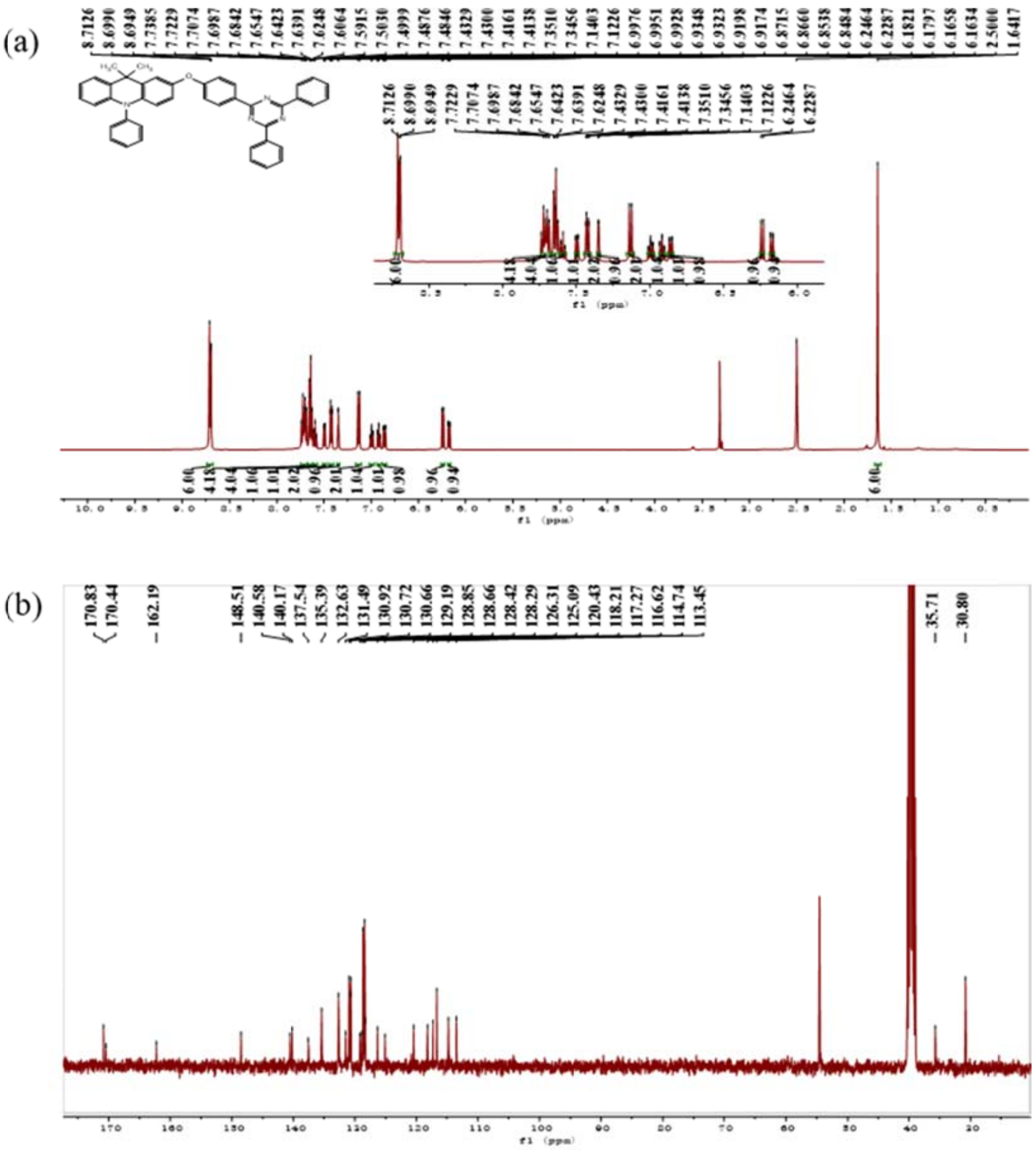

Supplementary Figure 4. ^{1}H (a) and ^{13}C NMR spectra (b) of RTP-D2.

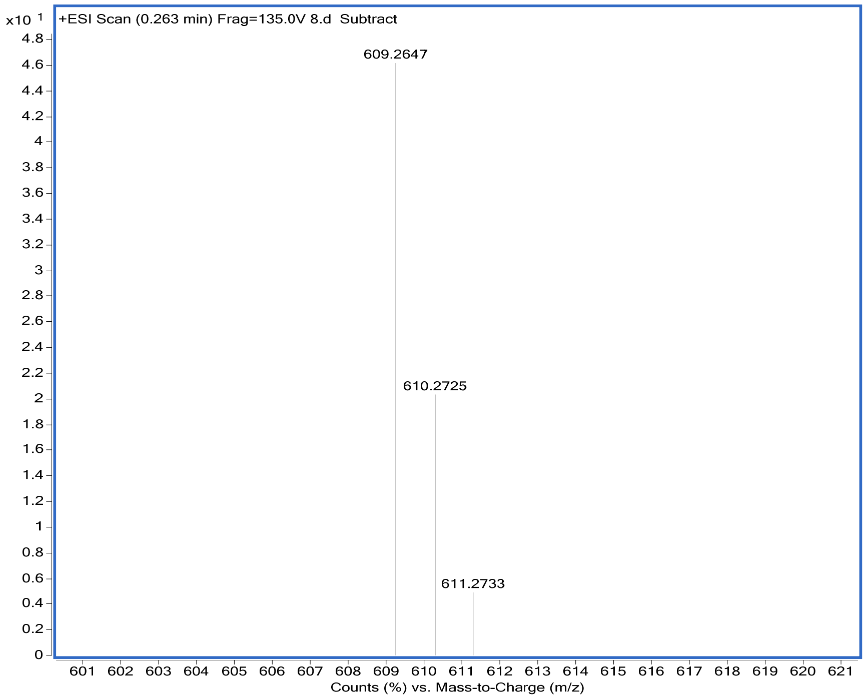

Supplementary Figure 5. ESI-MS spectrum of RTP-D2.

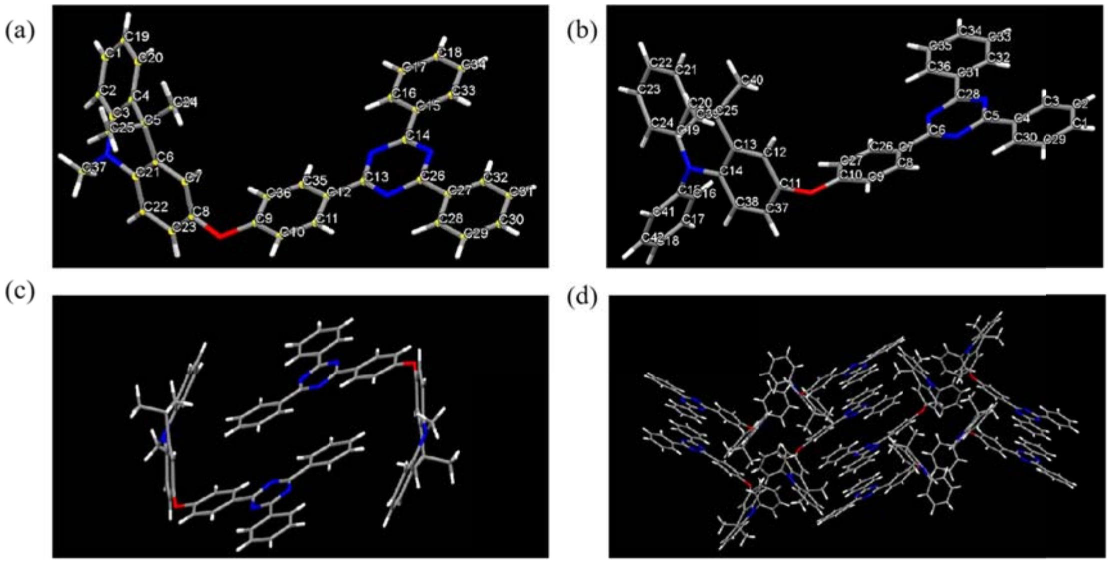

Supplementary Figure 6. Single crystal structures and packing modes of RTP-D1 (a and c) and RTP-D2 (b and d).

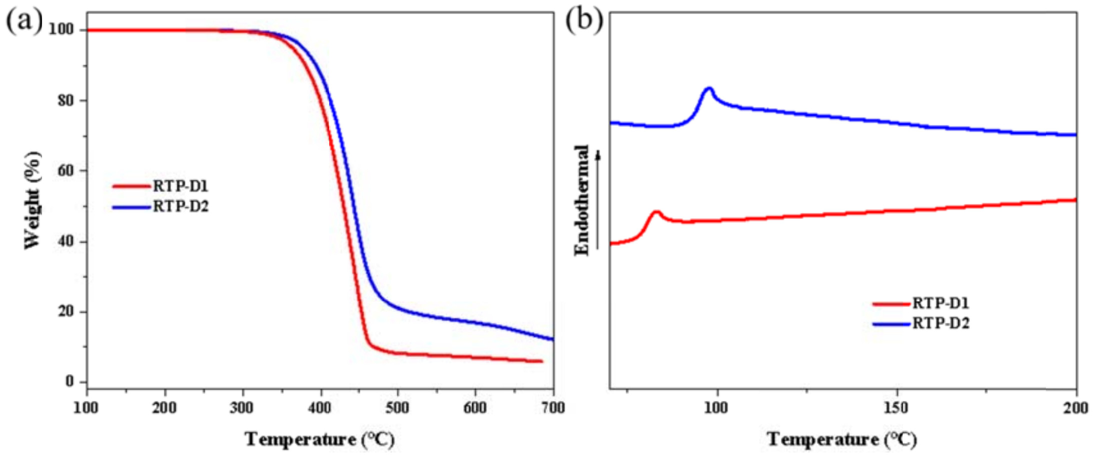

Supplementary Figure 7. TGA (a) and DSC traces (b) for RTP-D1 and RTP-D2.

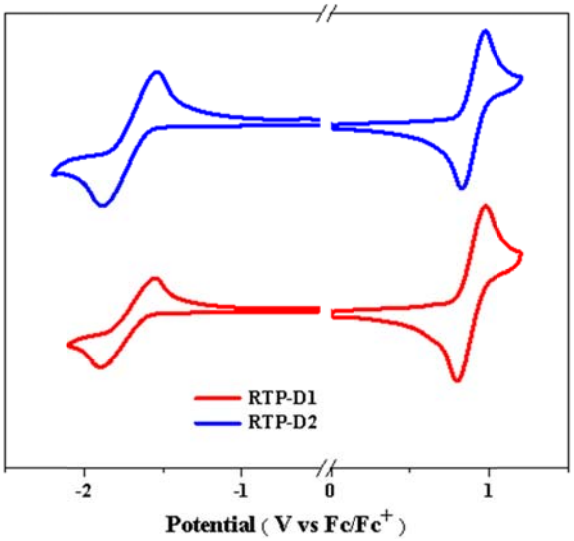

Supplementary Figure 8. CV curves of RTP-D1 and RTP-D2.

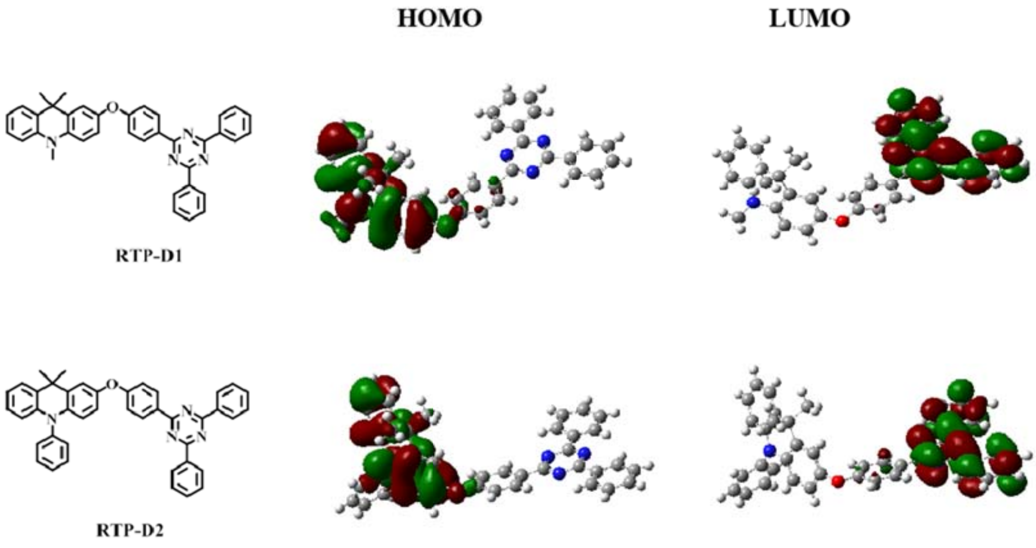

Supplementary Figure 9. Calculated HOMO and LUMO distributions for RTP-D1 and RTP-D2.

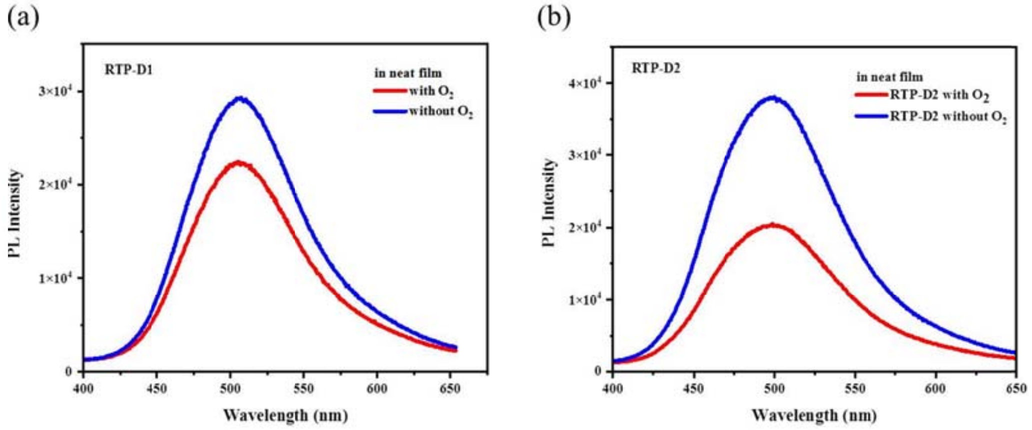

Supplementary Figure 10. The O_2 dependence of the PL spectra in neat films for RTP-D1 (a) and RTP-D2 (b). The PL spectra of RTP-D1 and RTP-D2 are found to be sensitive to O_2, indicative of the triplet exciton origin to some degree.

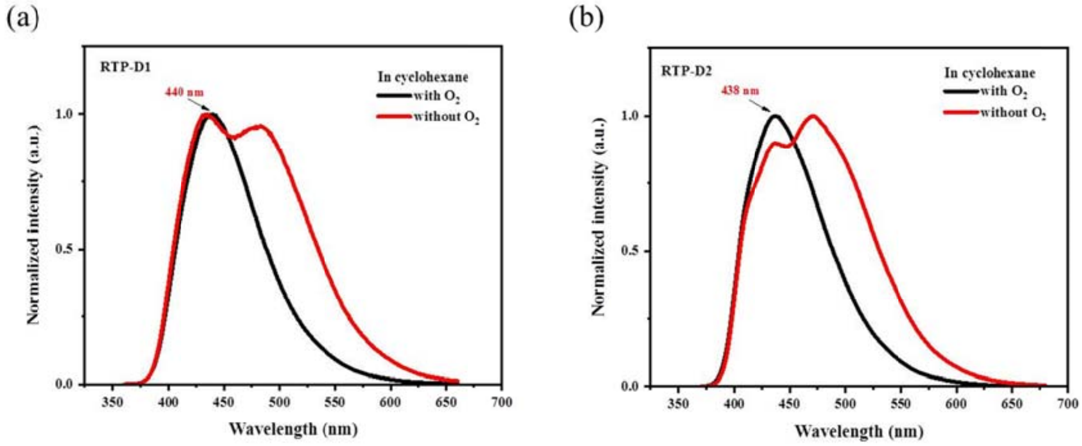

Supplementary Figure 11. The O_2 dependence of the PL spectra in cyclohexane (10^{-5} mol L^{-1}) for RTP-D1 (a) and RTP-D2 (b). As one can see, both RTP-D1 and RTP-D2 show two distinct emission bands in the absence of O_2. Assumed that the emission corresponding to the triplet excitons could be completely quenched by O_2 in dilute solution, the long-wavelength band is reasonably from the triplet exciton related RTP.

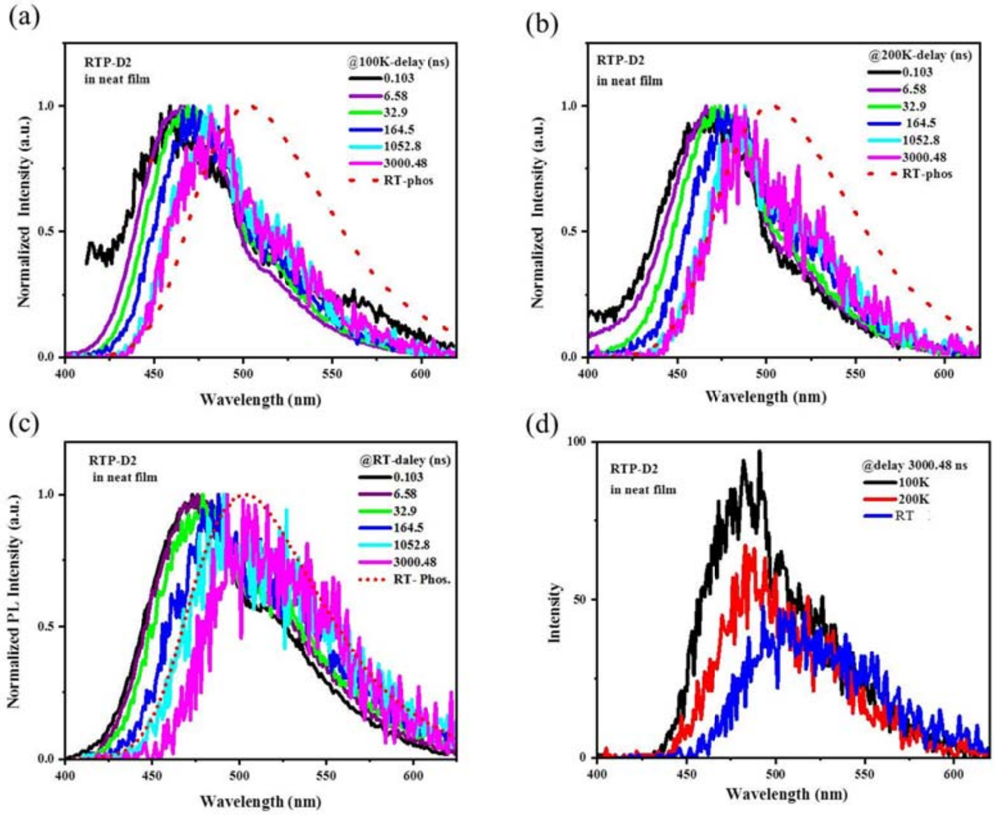

Supplementary Figure 12. TRES for the RTP-D2 film: (a) at 100 K; (b) at 200 K; (c) at room temperature; (d) Intensity comparison of the curves at a delay time of 3000.48 ns.

(a)

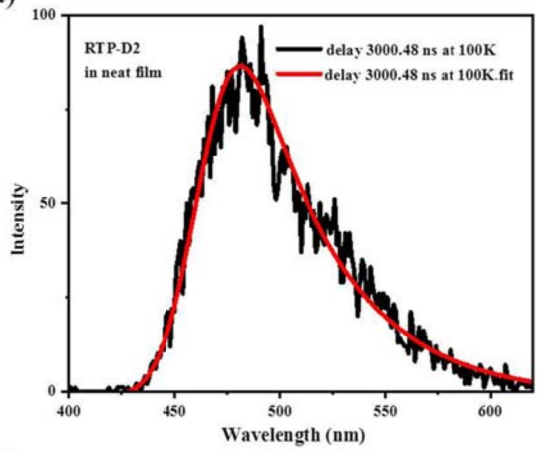

Model	GaussMod
Equation	double z = (x-xc)/w - w/t0; y = y0 + A/t0 * exp(0.5*(w/t0)^2-(x-xc)/t0)*(erf(z/sqrt(2))+1)/2;
Plot	Book1_G@2
y0	-1.38812 ± 0.46911
A	6583.41471 ± 130.86083
xc	463.60931 ± 0.61163
w	14.96108 ± 0.52325
t0	41.48071 ± 1.27549
Reduced Chi-Sqr	11.27361
R-Square (COD)	0.99816
Adj. R-Square	0.99813

(b)

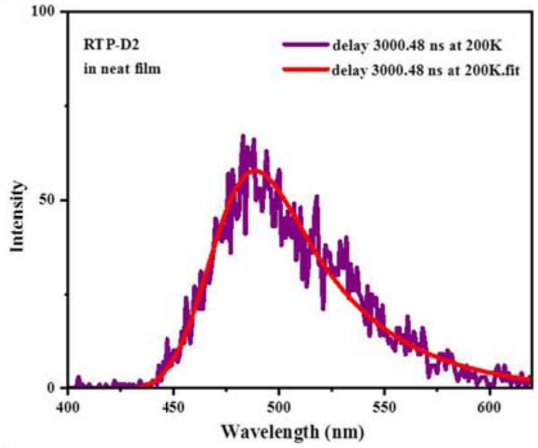

Model	GaussMod
Equation	double z = (x-xc)/w - w/t0; y = y0 + A/t0 * exp(0.5*(w/t0)^2-(x-xc)/t0)*(erf(z/sqrt(2))+1)/2;
Plot	Book2_G@2
y0	-0.64963 ± 0.4723
A	4384.74097 ± 122.95071
xc	470.78113 ± 0.8005
w	15.55112 ± 0.70161
t0	40.41333 ± 1.83015
Reduced Chi-Sqr	12.78582
R-Square (COD)	0.99776
Adj. R-Square	0.99772

(c)

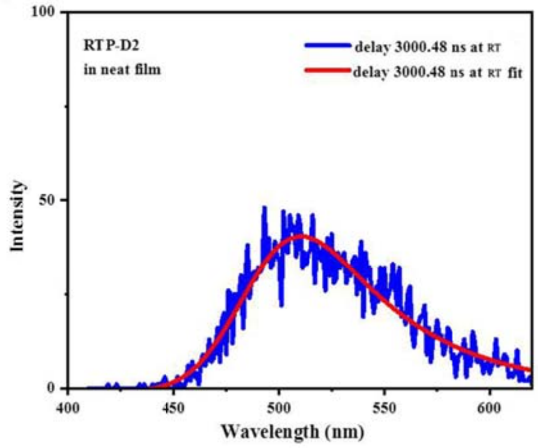

Model	GaussMod
Equation	double z = (x-xc)/w - w/t0; y = y0 + A/t0 * exp(0.5*(w/t0)^2-(x-xc)/t0)*(erf(z/sqrt(2))+1)/2;
Plot	Book5_G@2
y0	-0.85858 ± 0.63363
A	3872.2235 ± 163.99213
xc	487.74905 ± 1.24278
w	20.89925 ± 1.13815
t0	47.57558 ± 3.06448
Reduced Chi-Sqr	14.95892
R-Square (COD)	0.99657
Adj. R-Square	0.99651

Supplementary Figure 13. GaussMod fitting for the TRES collected at 3000.48 ns: (a) at 100 K; (b) at 200 K; (c) at room temperature.

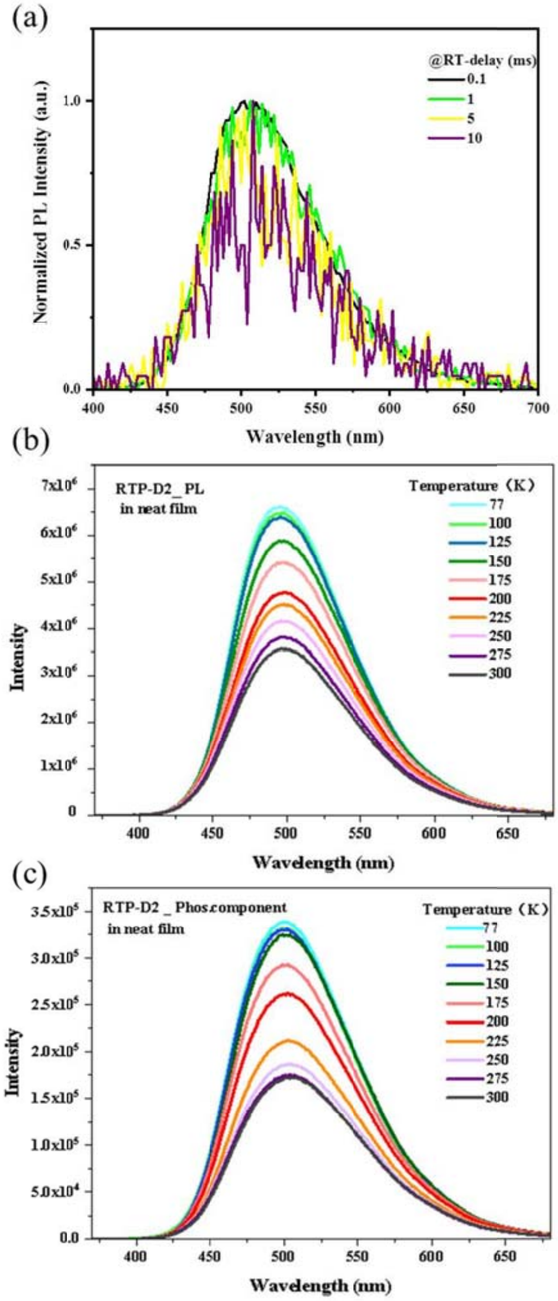

Supplementary Figure 14. (a) PL spectra at different delay times for the RTP-D2 film; (b) Temperature-dependent PL spectra (without a delay) for the RTP-D2 film; (c) Temperature-dependent phosphorescence spectra (with a 0.1 ms delay) for the RTP-D2 film. As one can see, the PL profile is nearly independent on the delay time. So a 0.1 ms delay is used for the measurement of phosphorescence spectra.

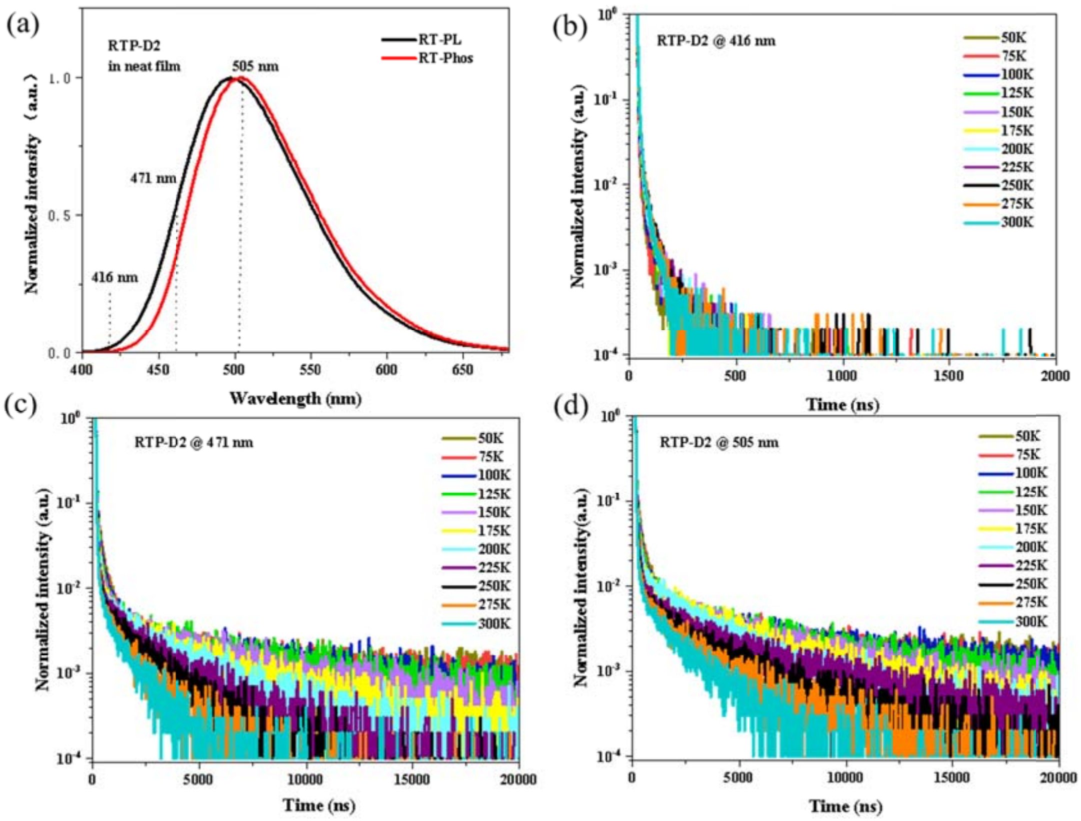

Supplementary Figure 15. PL and RTP spectra (a), and temperature-dependent transient PL spectra detected at a wavelength of 416 nm (b), 471 nm (c) and 505 nm (d) for RTP-D2 in neat film.

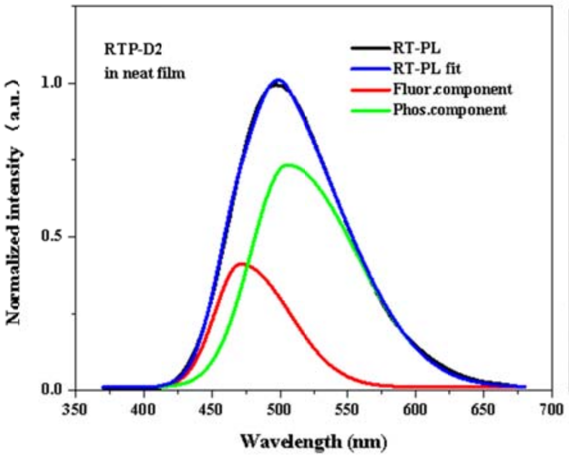

Model	Bigaussian	
Equation	if (x < xc) y = y0 + H*exp(-(x - xc)^2/(2*w1^2)); else	
Plot	Peak1(Normalized1	Peak2(Normalized1
y0	0.01037 ± 0.00104	0.01037 ± 0.0010
xc	471.21212 ± 0.643	505.3429 ± 0.822
H	0.40767 ± 0.01683	0.72024 ± 0.0167
w1	18.78636 ± 0.3270	26.47313 ± 0.408
w2	35.44948 ± 1.8229	50.82754 ± 0.678
Reduced Chi-Sqr	7.805E-5	
R-Square (COD)	0.99936	
Adj. R-Square	0.99935	

Supplementary Figure 16. Bigaussian fitting of the steady-state PL spectra for the RTP-D2 film. Given that fluorescence is dominated in the TRES spectra at the beginning, the peak of TRES spectrum at 0.103 ns (471 nm) is set as the maximum fluorescent emission. Combined with the maximum phosphorescent emission (505 nm) shown in the RTP spectrum, a Bigaussian fitting is performed to divide the fluorescence and RTP. By comparing their corresponding integral area, the populations of fluorescence and RTP are taken to be 29.4% and 70.6%, respectively.

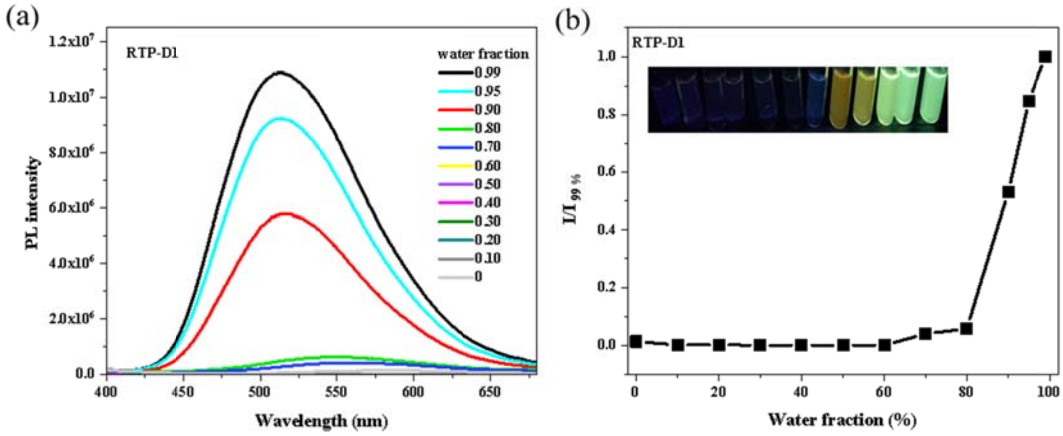

Supplementary Figure 17. AIE behavior of RTP-D1 in water/THF mixed solvents: (a) Dependence of the PL spectra on the water fraction; (b) Relative emission intensity as a function of water fraction. Insets: PL images under UV light with the increasing water fraction from left to right.

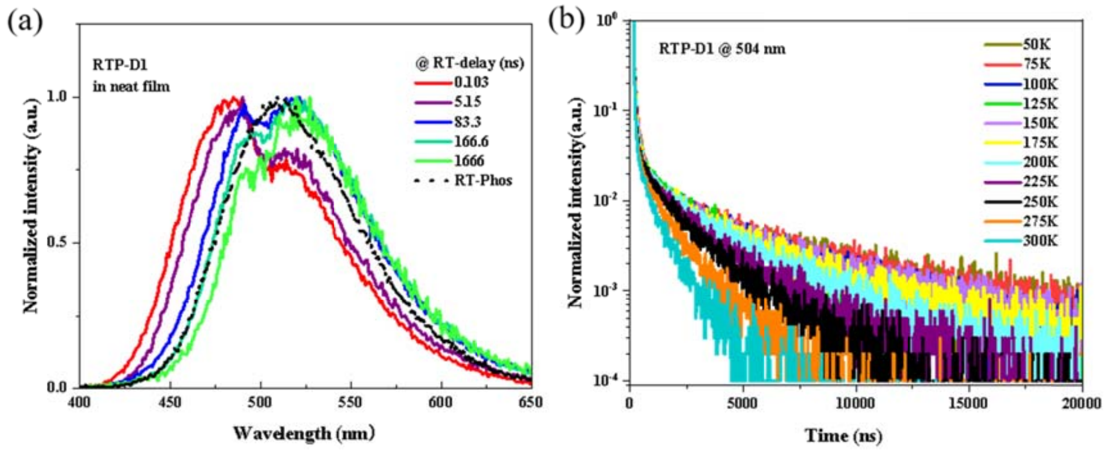

Supplementary Figure 18. RTP nature of RTP-D1 in neat film: (a) Time-resolved emission spectra compared with the corresponding phosphorescent spectrum at room temperature; (b) Temperature-dependent transient PL spectra detected at 504 nm.

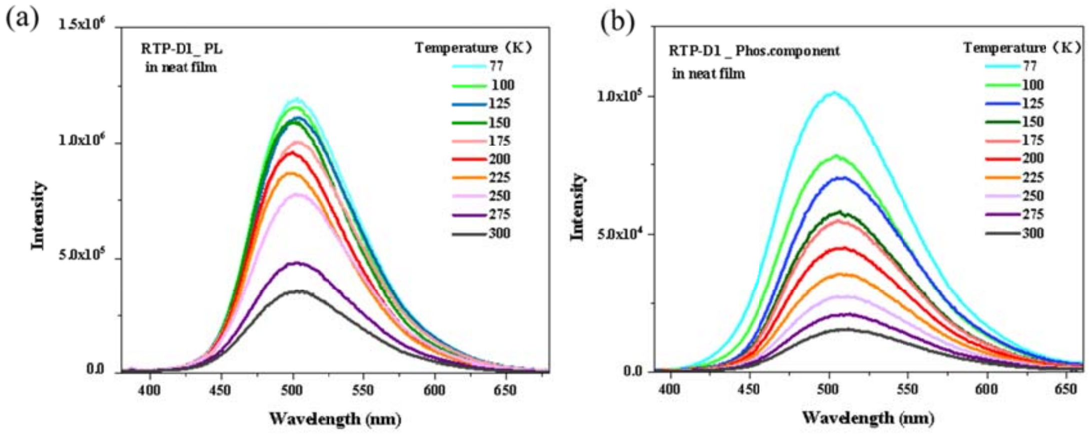

Supplementary Figure 19. (a) Temperature-dependent PL spectra (without a delay) for the RTP-D1 film; (b) Temperature-dependent phosphorescence spectra (with a 0.1 ms delay) for the RTP-D1 film.

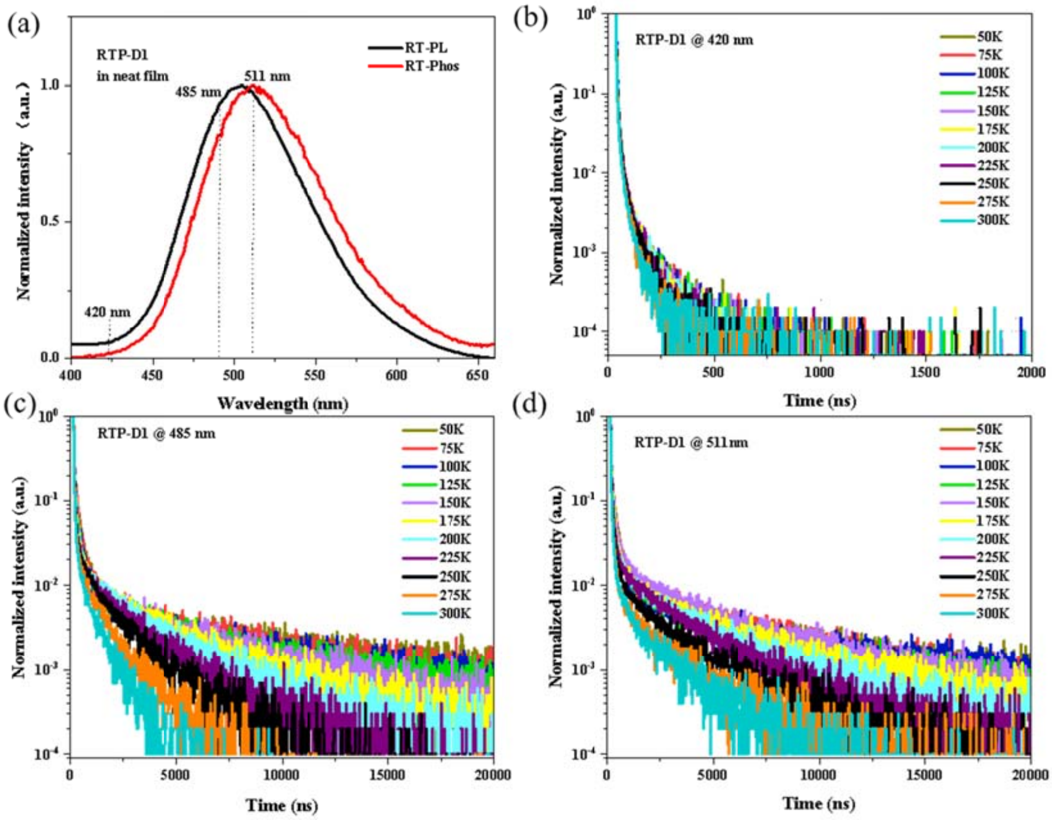

Supplementary Figure 20. PL and RTP spectra (a), and temperature-dependent transient PL spectra detected at a wavelength of 420 nm (b), 485 nm (c) and 511 nm (d) for RTP-D1 in neat film. In the case of 420 nm (the RTP contribution is negligible), no delayed component is observed. This clearly indicates that TADF is not involved in RTP-D1.

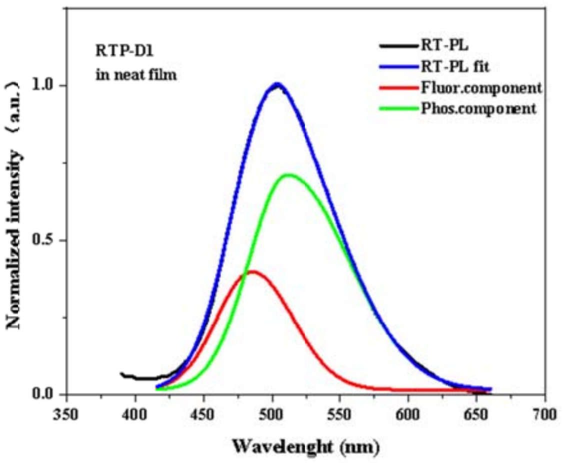

Model	Bigaussian	
Equation	if (x < xc)<br>y = y0 + H*exp(-(x - xc)^2/(2*w1^2));<br>else<br>y = y0 + H*exp(-(x - xc)^2/(2*w2^2));	
Plot	Peak1(DfltEm2_B@2)	Peak2(DfltEm2_B@2)
y0	0.03209 ± 0.00147	0.03209 ± 0.00147
xc	485.4067 ± 0	511.563 ± 0
H	0.3175 ± 0.01348	0.7377 ± 0.01505
w1	22.27893 ± 0.60617	30.55691 ± 0.50287
w2	26.76034 ± 1.29237	42.36992 ± 0.41085
Reduced Chi-Sqr	1.48378E-4	
R-Square (COD)	0.99873	
Adj. R-Square	0.9987	

Supplementary Figure 21. Bigaussian fitting of the steady-state PL spectra for the RTP-D1 film. Given that fluorescence is dominated in the TRES spectra at the beginning, the peak of TRES spectrum at 0.103 ns (485 nm) is set as the maximum fluorescent emission. Combined with the maximum phosphorescent emission (511 nm) shown in the RTP spectrum, a Bigaussian fitting is performed to divide the fluorescence and RTP. By comparing their corresponding integral area, the populations of fluorescence and RTP are taken to be 33.1% and 66.9%, respectively.

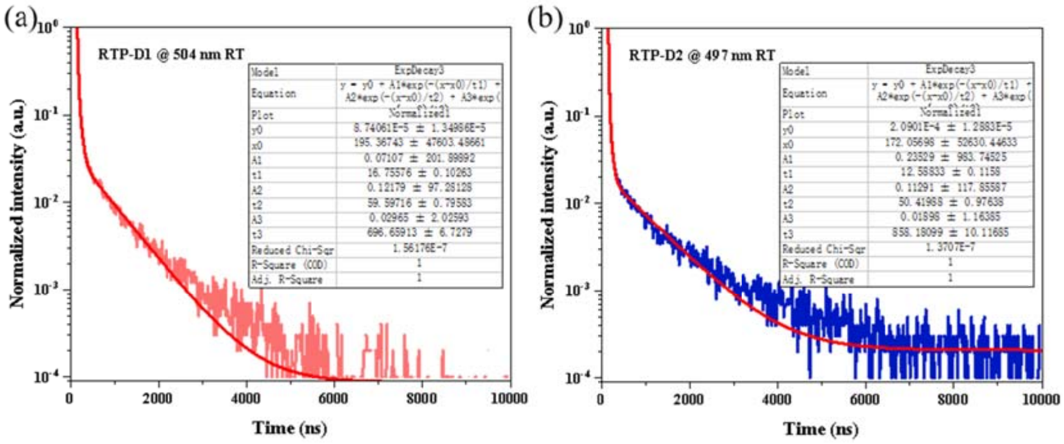

Supplementary Figure 22. Transient PL spectra in neat films for RTP-D1 (a) and RTP-D2 (b).

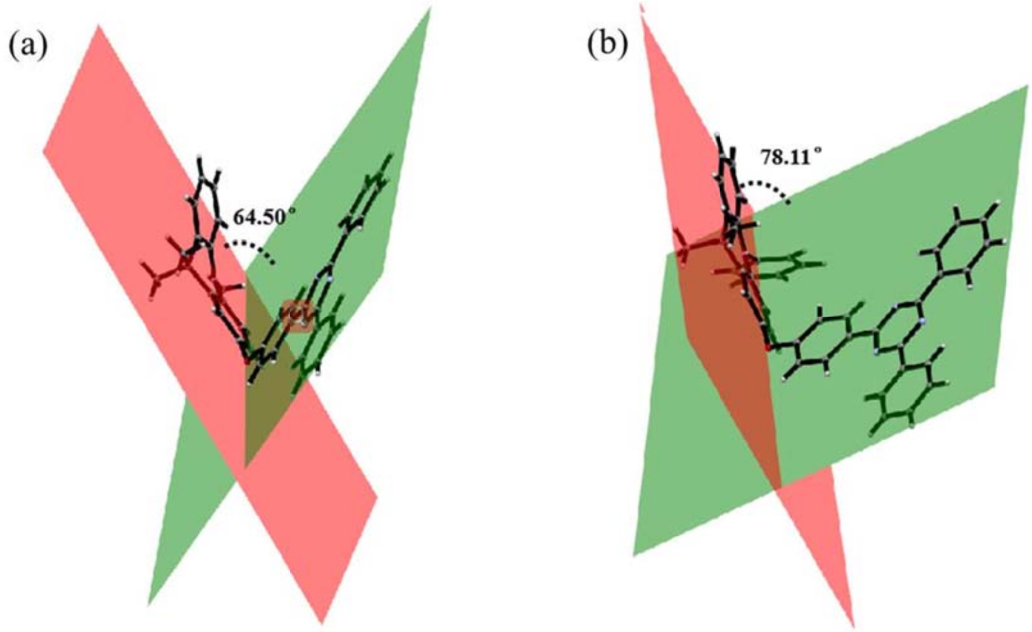

Supplementary Figure 23. Dihedral angle between the acridine donor and the triazine acceptor for RTP-D1 (a) and RTP-D2 (b).

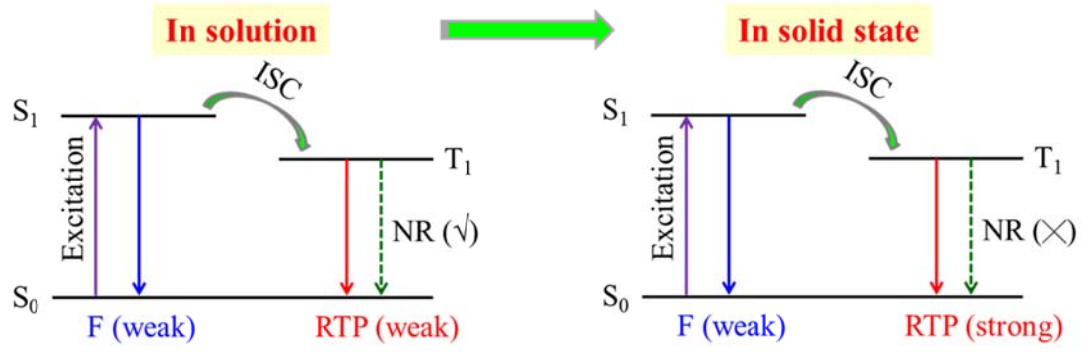

Supplementary Figure 24. Schematic illustration of aggregation-induced RTP for D-O-A based organic phosphors.

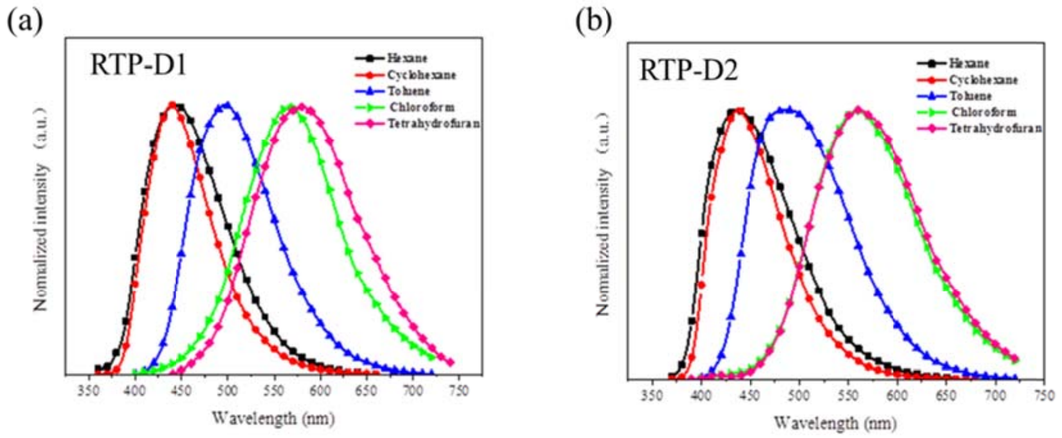

Supplementary Figure 25. PL spectra measured in different organic solvents in the presence of O_2 for RTP-D1 (a) and RTP-D2 (b).

When the water fraction grows up, it should be noted that the PL spectra are also show a hypsochromic shift besides the enhanced emission intensity (Figure 3 and S16). As discussed above, the PL in solution with O_2 is corresponding to the fluorescence, because the weak RTP is completely quenched by O_2. Due to the charge transfer (CT) nature, fluorescence is sensitive to the solvent polarity. So the emission maxima of RTP-D1 and RTP-D2 appear at about 575 nm in THF. However, in aggregates, the corresponding PL is dominantly from RTP peaked at about 500 nm. Therefore, the PL spectra are found to be blue-shifted with the increasing water fraction.

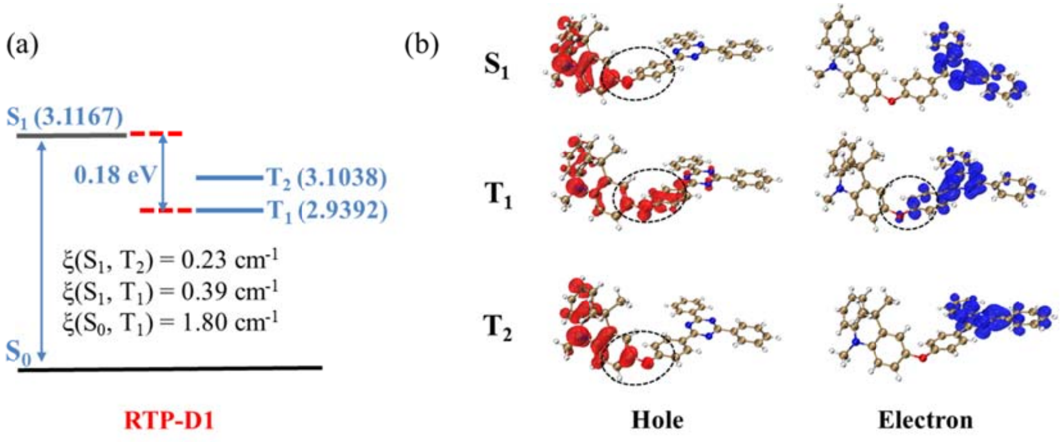

Supplementary Figure 26. Energy level alignment together with the spin-orbit coupling matrix elements (a), and hole and electron distributions of S_1 and T_n below S_1 for RTP-D1 (b).

Compared with S_1, it is found that the hole of T_1 is extended from acridine and O to triazine, and the electron of T_1 is extended from triazine to O. During the hole and electron migrations from S_1 to T_1, both the n orbital contribution and the large dihedral angle between D and A (64.50^o for RTP-D1 in Figure S22) plays an important role on the orbital angular momentum change, which can compensate the spin angular momentum. Therefore, the spin flipping becomes more allowed from S_1 to T_1 than from S_1 to T_2.

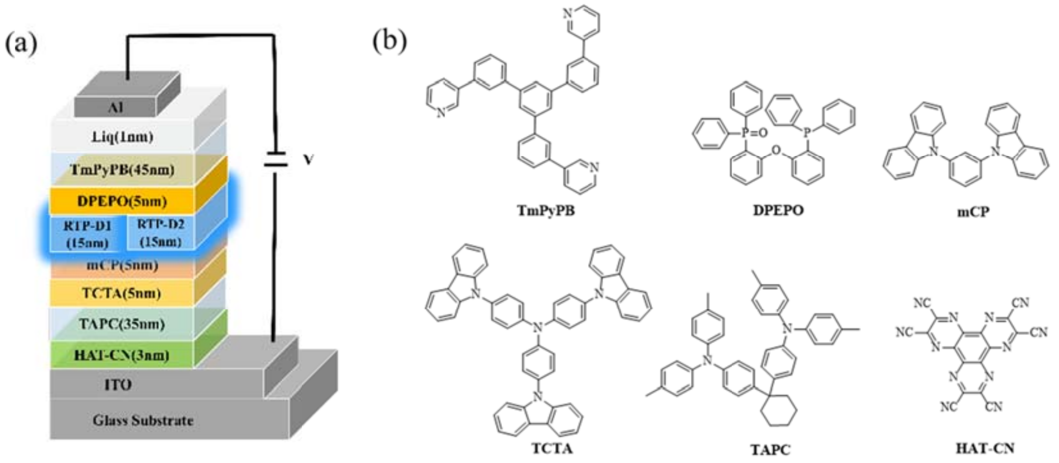

Supplementary Figure 27. Device configuration (a) and molecular structures of used materials (b).

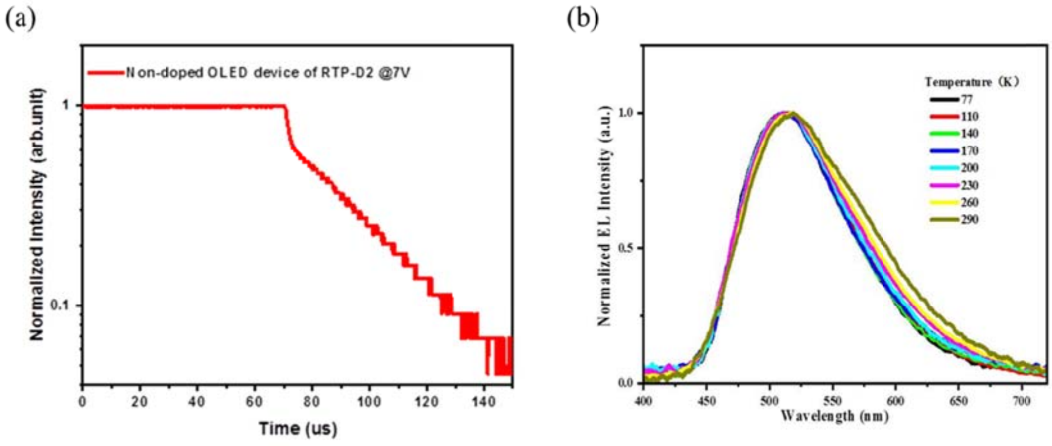

Supplementary Figure 28. Transient EL spectrum driven at a 7 V pulse (a) and temperature dependence of the EL spectra (b) for RTP-D2 based non-doped device. After switching off the electrical pulse, an obvious delay is observed in the transient EL, well consistent with the transient PL.

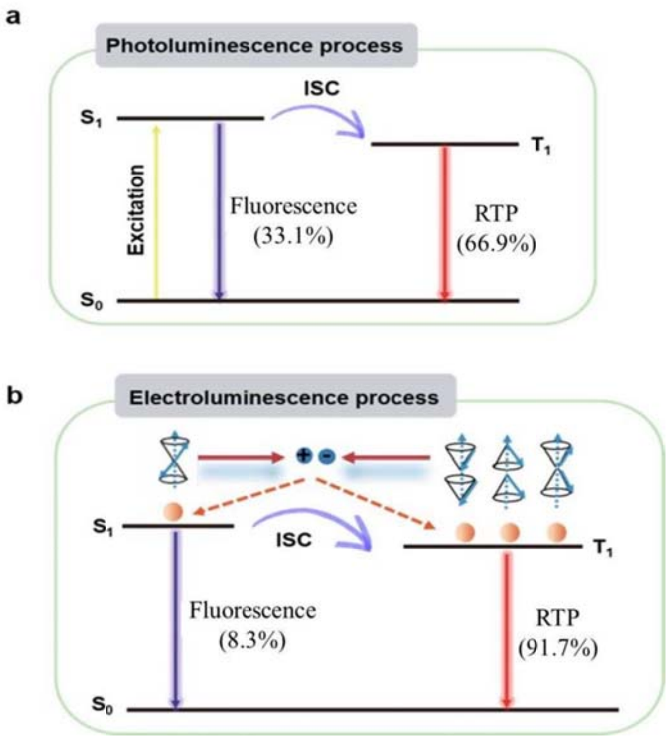

Supplementary Figure 29. Comparison between the PL (**a**) and EL (**b**) processes for RTP-D1. First, the Bigaussian fitting decouples 33.1% fluorescence from singlet excitons and 66.9% RTP from triplet excitons (Figure S16). Second, under photo excitation, triplet excitons must be produced only through ISC. So the ISC probility from S_1 to T_1 is reasonably set to be the same as that of the RTP population (66.9%). Third, under electric excitation, 25% singlet and 75% triplet excitons are often generated from the injected holes and electrons. Assuming a same ISC probability as the PL process, 25% singlet excitons can spin-flip to form 16.7% triplet excitons (25% × 66.9%). In all, a total of 91.7% (16.7% + 75%) triplet excitons contribute to the EL.

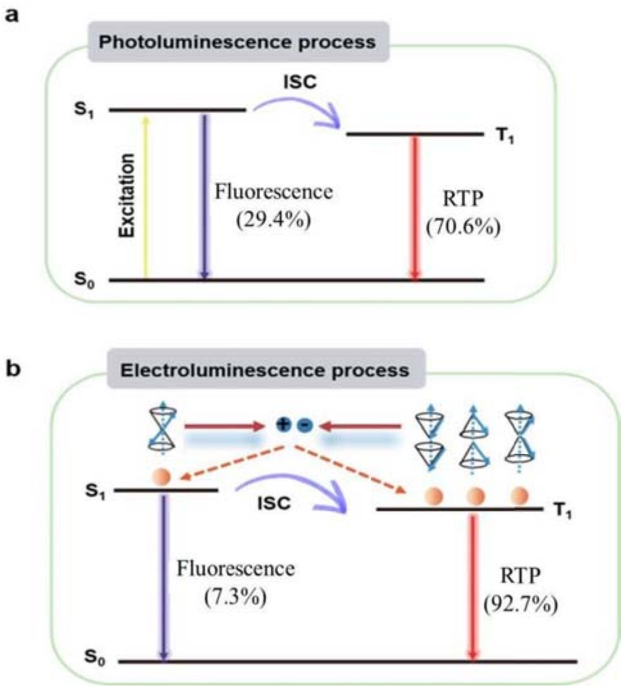

Supplementary Figure 30. Comparison between the PL (**a**) and EL (**b**) processes for RTP-D2. First, the Bigaussian fitting decouples 29.4% fluorescence from singlet excitons and 70.6% RTP from triplet excitons (Figure S11). Second, under photo excitation, triplet excitons must be produced only through ISC. So the ISC probility from S_1 to T_1 is reasonably set to be the same as that of the RTP population (70.6%). Third, under electric excitation, 25% singlet and 75% triplet excitons are often generated from the injected holes and electrons. Assuming a same ISC probability as the PL process, 25% singlet excitons can spin-flip to form 17.7% triplet excitons (25% × 70.6%). In all, a total of 92.7% (17.7% + 75%) triplet excitons contribute to the EL.

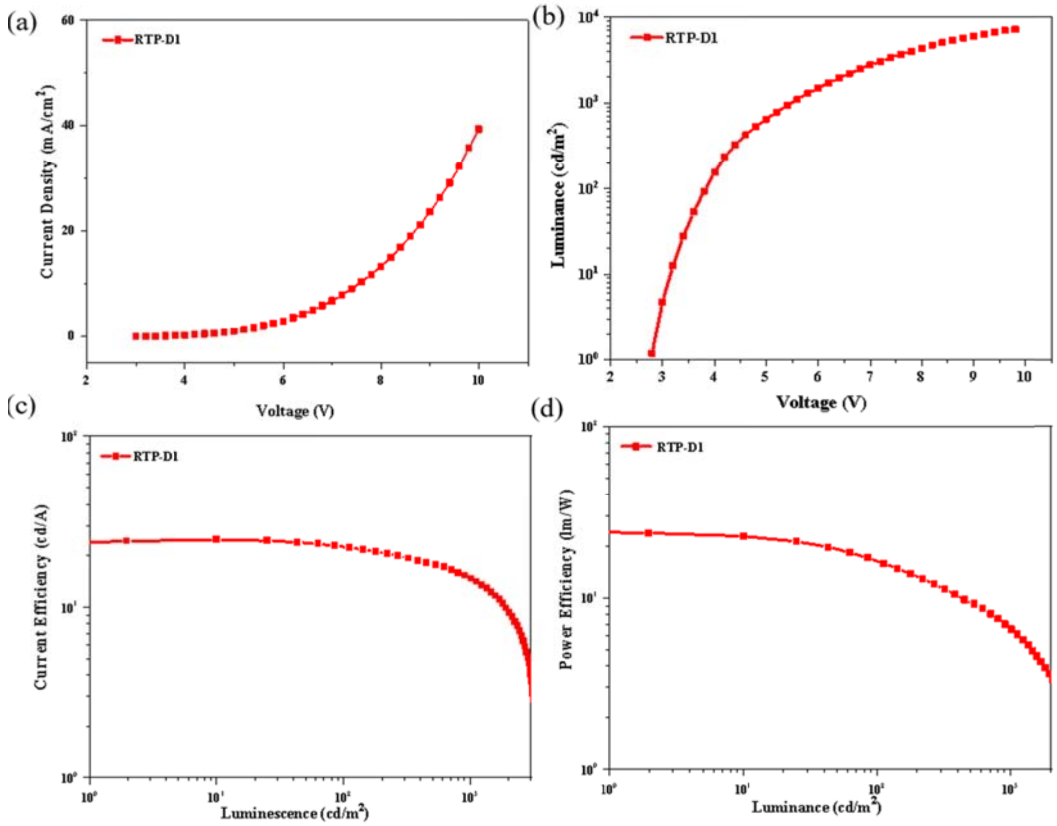

Supplementary Figure 31. Non-doped device performance of RTP-D1: (a) Current density–voltage characteristic; (b) Luminance–voltage characteristic; (c) Current efficiency as a function of luminance; (d) Power efficiency as a function of luminance.

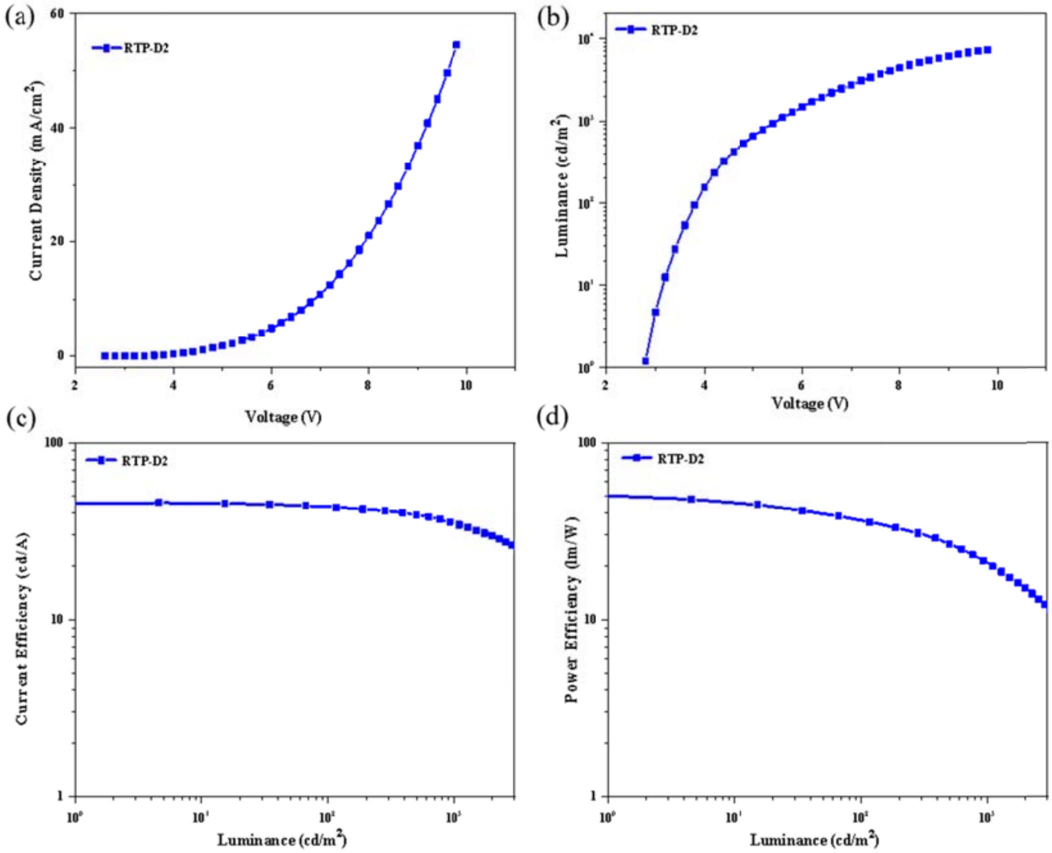

Supplementary Figure 32. Non-doped device performance of RTP-D2: (a) Current density–voltage characteristic; (b) Luminance–voltage characteristic; (c) Current efficiency as a function of luminance; (d) Power efficiency as a function of luminance.

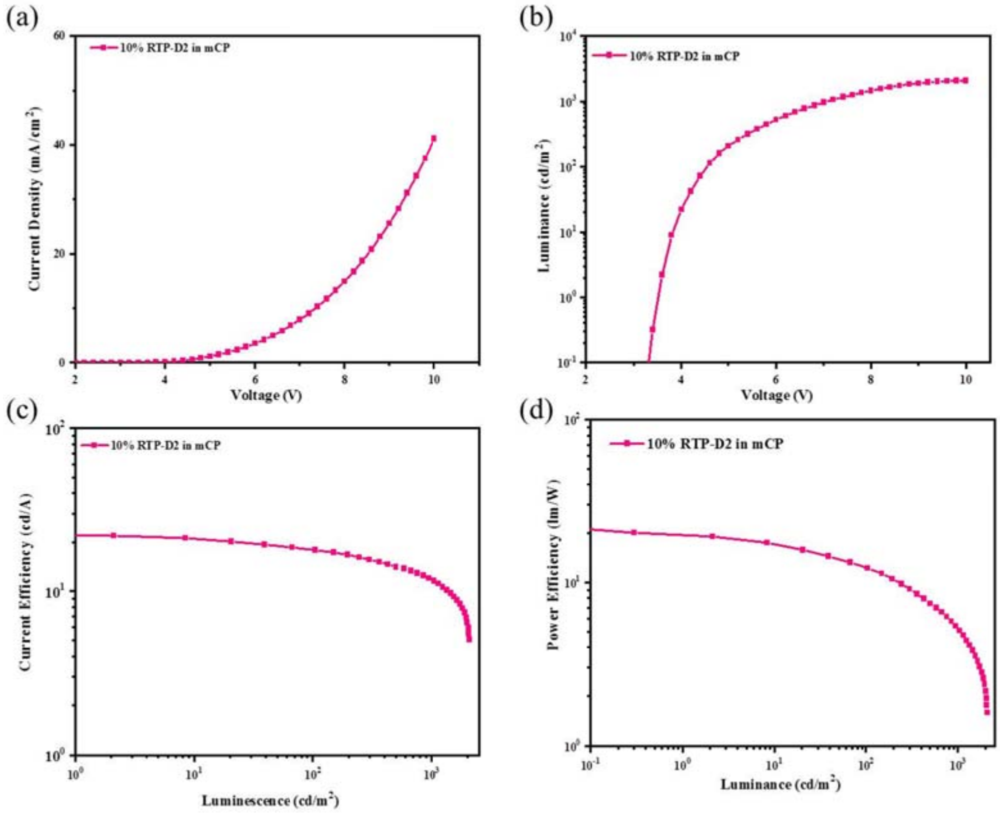

Supplementary Figure 33. Doped device performance of 10% RTP-D2 in mCP: (a) Current density–voltage characteristic; (b) Luminance–voltage characteristic; (c) Current efficiency as a function of luminance; (d) Power efficiency as a function of luminance.

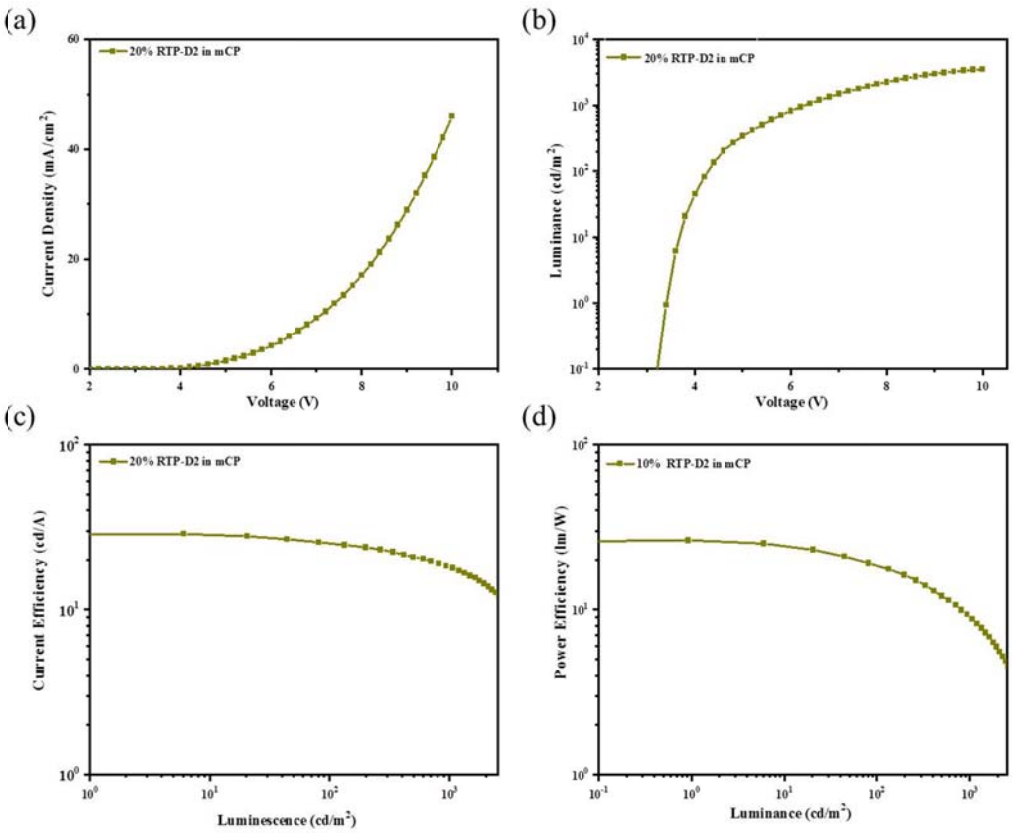

Supplementary Figure 34. Doped device performance of 20% RTP-D2 in mCP: (a) Current density–voltage characteristic; (b) Luminance–voltage characteristic; (c) Current efficiency as a function of luminance; (d) Power efficiency as a function of luminance.

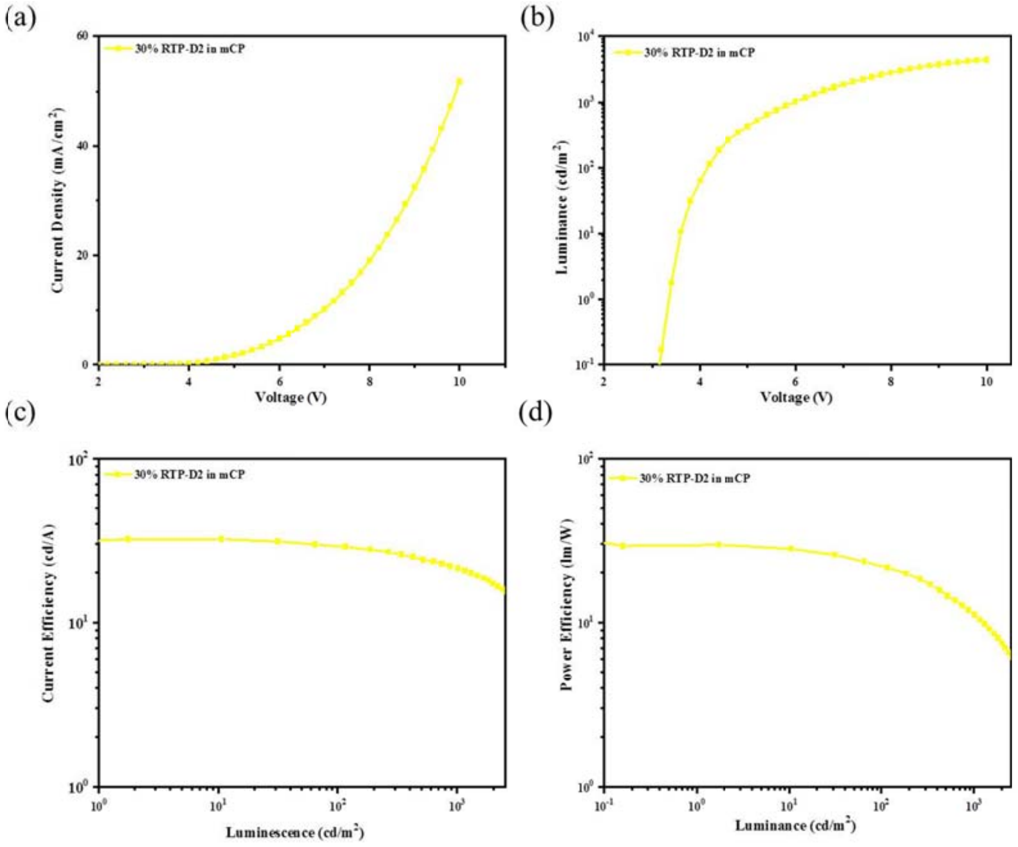

Supplementary Figure 35. Doped device performance of 30% RTP-D2 in mCP: (a) Current density–voltage characteristic; (b) Luminance–voltage characteristic; (c) Current efficiency as a function of luminance; (d) Power efficiency as a function of luminance.

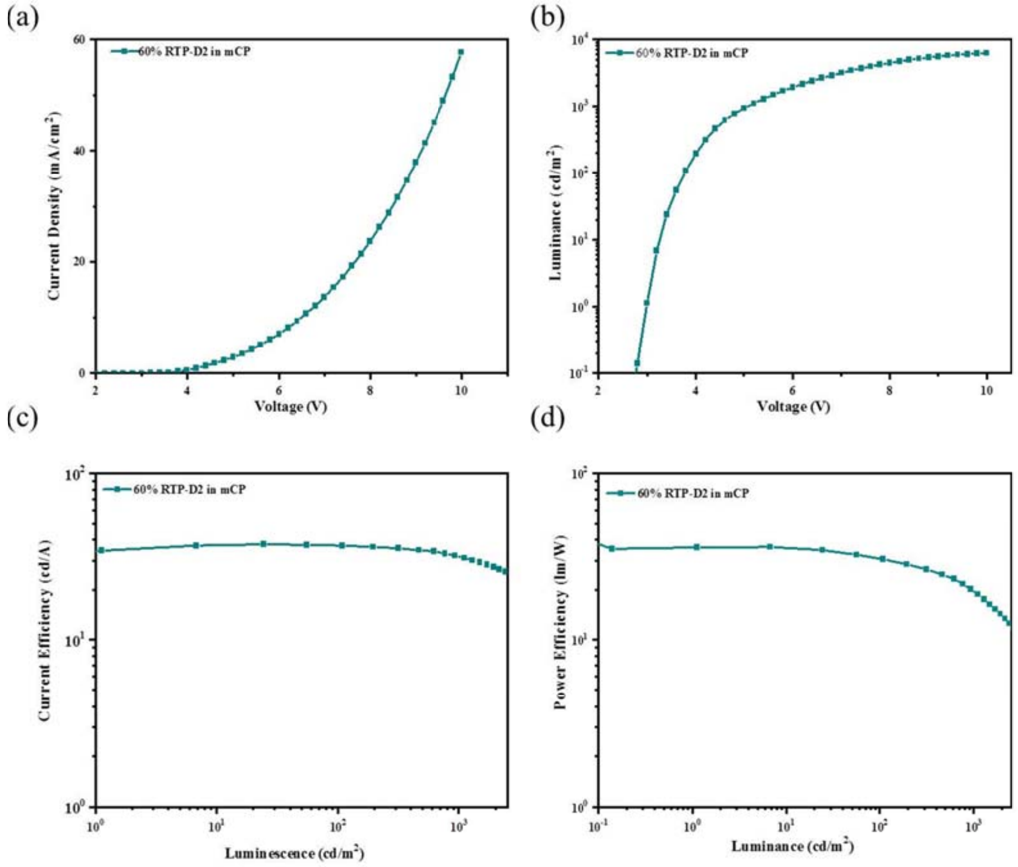

Supplementary Figure 36. Doped device performance of 60% RTP-D2 in mCP: (a) Current density–voltage characteristic; (b) Luminance–voltage characteristic; (c) Current efficiency as a function of luminance; (d) Power efficiency as a function of luminance.

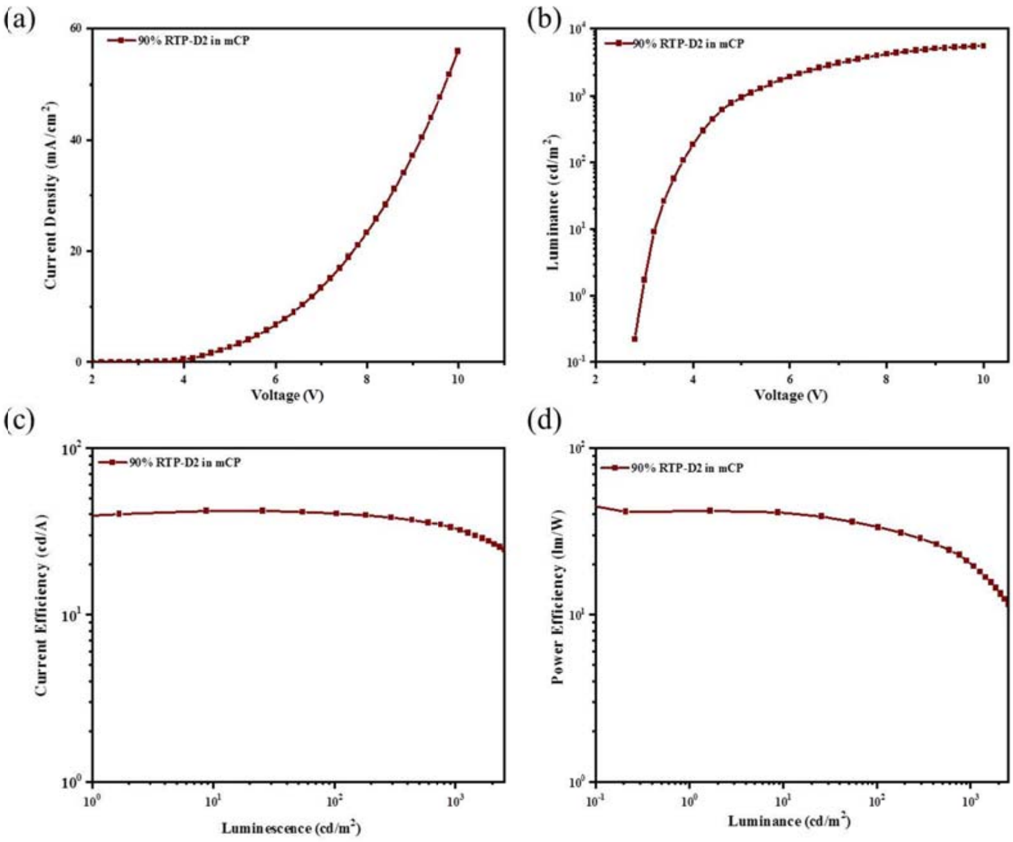

Supplementary Figure 37. Doped device performance of 90% RTP-D2 in mCP: (a) Current density–voltage characteristic; (b) Luminance–voltage characteristic; (c) Current efficiency as a function of luminance; (d) Power efficiency as a function of luminance.

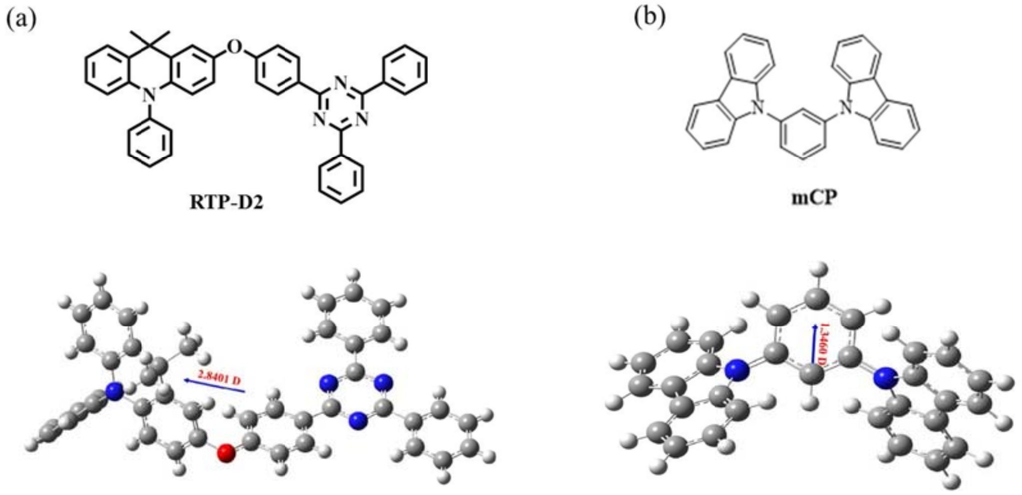

Supplementary Figure 38. Dipole moment for RTP-D2 (a) and mCP (b). According to the theoretical calculation, the dipole moment is estimated to be 2.8401 D and 1.3460 D for RTP-D2 and mCP, respectively.

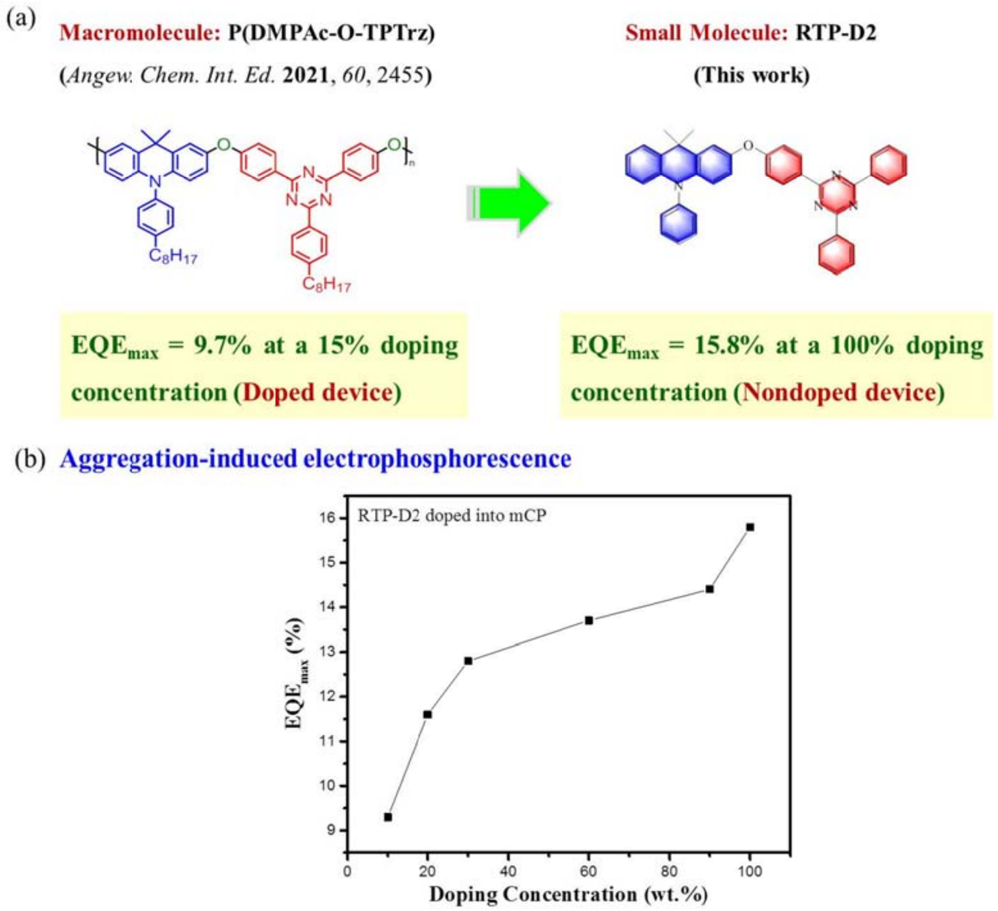

Supplementary Figure 39. (a) Comparison between D-O-A based polymer and small molecule; (b) Maximum EQE as a function of doping concentration for RTP-D2 in mCP.

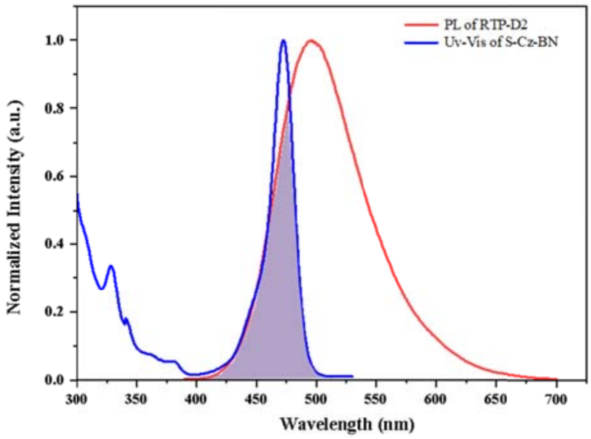

Supplementary Figure 40. Overlap between the absorption of S-Cz-BN and the PL of RTP-D2.

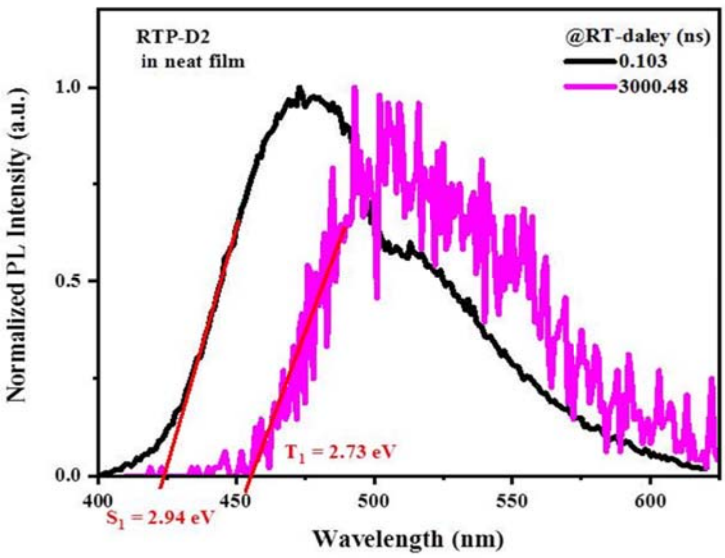

Supplementary Figure 41. Determination of the energy levels of S_1 and T_1 for RTP-D2. According to the TRES analysis, fluorescence and phosphorescence dominate the PL at a delay time of 0.103 ns and 3000.48 ns, respectively. Therefore, their onset values are reasonably taken as the S_1 and T_1 energies of RTP-D2.

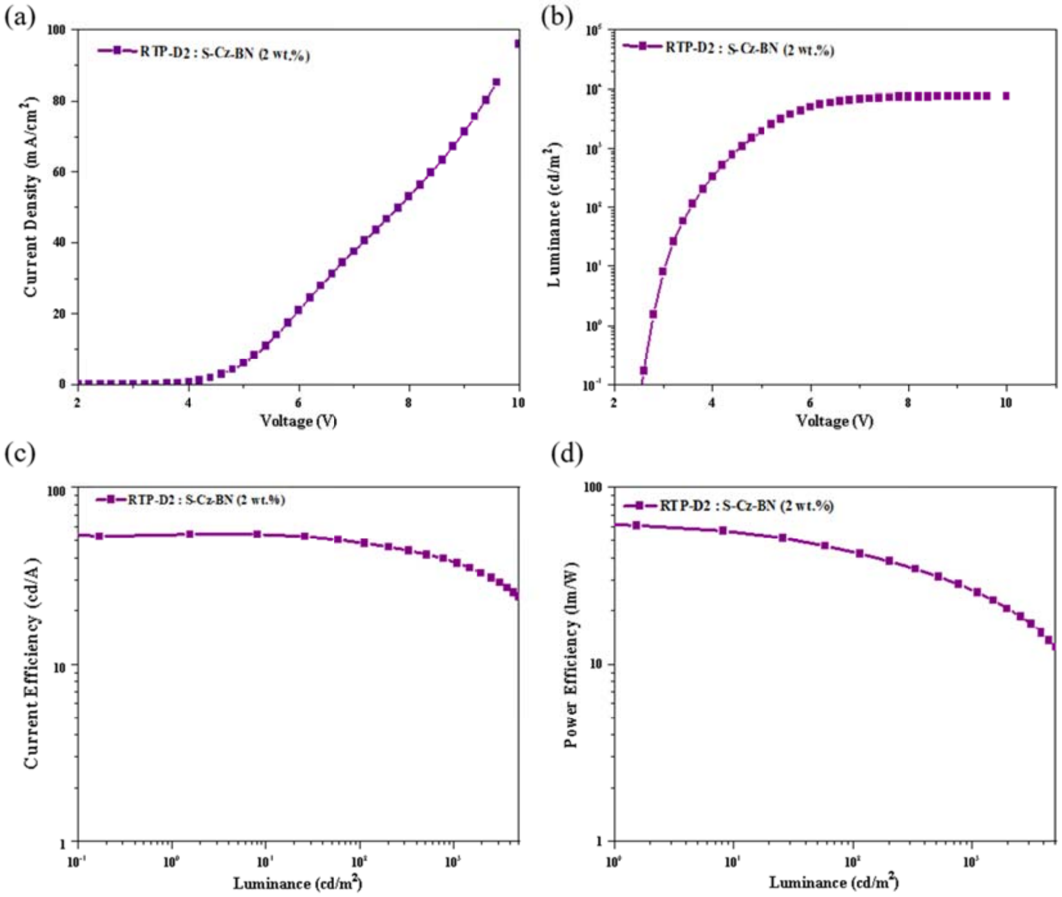

Supplementary Figure 42. Device performance of RTP-D2 : S-Cz-BN (2 wt.%) based host-free sensitization: (a) Current density–voltage characteristic; (b) Luminance–voltage characteristic; (c) Current efficiency as a function of luminance; (d) Power efficiency as a function of luminance.

Supplementary Tables

Supplementary Table 1. X-Crystal data and structure refinement for RTP-D1 and RTP-D2.

	RTP-D1	RTP-D2
Empirical formula	$C_{37}H_{30}N_4O$	$C_{42}H_{32}N_4O$
Formula weight	546.65	608.71
Temperature/K	150 K	150 K
Crystal system	Triclinic	Orthorhombic
Space group	P-1	Pbca
a/Å	7.9086 (13)	24.563 (5)
b/Å	13.696 (2)	7.4471 (16)
c/Å	14.996 (2)	34.619 (7)
α/°	63.761 (6)	90
β/°	79.572 (6)	90
γ/°	74.229 (7)	90
Volume/$Å^3$	1398.6 (4)	6333 (2)
Z	2	8
P_{calc} mg/cm^3	1.298	1.277
Absorption coefficient/mm	0.079	0.078
F(000)	576	2560
Crystal size/mm^3	0.34 x 0.28 x 0.19	0.32 x 0.3 x 0.14
Radiation	MoKα (0.71073 Å)	MoKα (0.71073 Å)
Theta range for data collection/°	2.683 to 28.391	2.033 to 24.998
Index ranges	-10<=h<=10, -18<=k<=18, -20<=l<=19	-24<=h<=29, -8<=k<=8, -41<=l<=31
Reflections collected	49202	31687
Independent reflections	7004 [R(int) = 0.0825]	5571 [R(int) = 0.1079]
Data/restraints/parameters	7004 / 0 / 382	5571 / 0 / 426
Goodness-of-fit on F^2	1.032	0.943
Final R indexes [I>=2σ (I)]	R_1 = 0.0491, wR_2 = 0.1268	R_1 = 0.0576, wR_2 = 0.1289
Final R indexes [all data]	R_1 = 0.0774, wR_2 = 0.1508	R_1 = 0.1154, wR_2 = 0.1562
Largest diff. peak/hole / e $Å^{-3}$	0.716 and -0.285	0.248 and -0.254

Supplementary Table 2. Photophysical properties and related parameters of RTP-D1 and RTP-D2.

Emitter	RTP-D1	RTP-D2
τ_F [ns]	16.8	12.6
F population [%] [a]	33.1	29.4
τ_P [ns]	488.8	641.4
RTP population [%] [a]	66.9	70.6
Φ_F [%] [b]	18.2	22.8
Φ_P [%] [b]	36.7	54.7
Φ_{PL} [%] [b]	54.9	77.5
k_{r}^F [10^7 S^{-1}] [c]	1.1	1.8
k_{nr}^F [10^8 S^{-1}] [c]	0.27	0.18
k_r^P [10^5 S^{-1}] [c]	7.5	8.5
k_{nr}^P [10^6 S^{-1}] [c]	1.3	0.7
k_{ISC} [10^7 S^{-1}] [c]	2.2	4.3

[a]Fluorescence (F) and room-temperature phosphorescence (RTP) populations were estimated from a Bigaussian fitting of the PL spectra; [b]The PLQYs of fluorescence (Φ_F) and RTP (Φ_P) were determined by the total PLQY (Φ_{PL}) and their corresponding populations; [c]The fluorescence radiative rate (k_{r}^F), fluorescence non-radiative rate (k_{nr}^F), phosphorescence radiative rate (k_r^P), phosphorescence non-radiative rate (k_{nr}^P) and intersystem crossing rate (k_{ISC}) were obtained by the following equations:

$$k_{\mathrm{r}}^F = \Phi_F / \tau_F \qquad k_{nr}^F = (1 - \Phi_F - \Phi_P) / \tau_F \qquad k_r^P = \Phi_P / \tau_P$$

$$k_{nr}^P = (1 - \Phi_P) / \tau_P \qquad k_{ISC} = \Phi_P / \tau_F$$

Supplementary Table 3. Device performance comparison of organic RTP emitters used for OLEDs.

Year	Emitter	EML	EQE_{max} [%]	CE_{max} [cd/A]	PE_{max} [lm/W]	Reference
2016	DMFLTPD-d_{36}	doped	1	-	-	*Adv. Mater.* **2016**, *28*, 655.
2018	OPM	doped	0.6	-	-	*Angew. Chem. Int. Ed.* **2018**, *57*, 16407.
2019	27PNDO	doped	0.11	-	-	*J. Mater. Chem. C* **2019**, *7*, 11500.
2019	BCZ1	nondoped	5.8	13.4	10.5	*Adv. Mater.* **2019**, *31*, 1904273.
2019	DPTZN	doped	11.5	33.8	32.6	*J. Phys. Chem. Lett.* **2019**, *10*, 5983.
2020	PSe1	doped	10.7	-	-	*Chem. Mater.* **2020**, *32*, 2583.
2020	BrPFL	doped	2.5	-	-	*ACS Appl. Mater. Interfaces* **2020**, *12*, 6137.
2021	SiAz	doped	4.06	-	-	*ACS Appl. Mater. Interfaces* **2021**, *13*, 2899.
2021	pBZ-DPA	doped	4.3	6.8	6.3	*Dyes and Pigments* **2021**, *195*, 109729.
2021	BuPhSe	doped	9.0	28.39	22.29	*J. Mater. Chem. C* **2021**, *9*, 8233.
2022	RTP-D1	nondoped	8.6	24.7	24.4	This work
	RTP-D2	nondoped	15.8	45.8	50.4	

CE: current efficiency; PE: power efficiency; EQE: external quantum efficiency.

Supplementary Table 4. Device performance dependence on the doping concentration of RTP-D2.

Doping ratio	V_{on} [a]	CE [b]	PE [b]	EQE [b]
10 %	3.4	22.0 /11.6	19.2 /5.1	9.3 /4.9
20 %	3.4	28.8 /17.9	25.1 /8.8	11.6 /7.0
30 %	3.4	32.2 /21.3	29.8 /11.2	12.8 /8.4
60 %	3	38.2 /30.1	36.0/18.8	13.7 /9.4
90 %	3	42.0 /32.2	42.0 /19.4	14.4 /11.0
100 %	2.8	45.8 /35.5	50.4 /21.4	15.8 /11.7

[a]Turn-on voltage at 1 cd/m^2; [b]Data at maximum and 1000 cd/m^2. CE: current efficiency; PE: power efficiency; EQE: external quantum efficiency.
